# OCT-angiography: Regional reduced macula microcirculation in ocular hypertensive and pre-perimetric glaucoma patients

**DOI:** 10.1371/journal.pone.0246469

**Published:** 2021-02-11

**Authors:** Bettina Hohberger, Marianna Lucio, Sarah Schlick, Antonia Wollborn, Sami Hosari, Christian Mardin

**Affiliations:** 1 Department of Ophthalmology, Friedrich-Alexander-University of Erlangen-Nürnberg, Erlangen, Germany; 2 Helmholtz Zentrum München - German Research Center for Environmental Health, Research Unit Analytical BioGeoChemistry, Neuherberg, Germany; National Eye Centre, UNITED STATES

## Abstract

**Purpose:**

OCT-angiography (OCT-A) offers a non-invasive method to visualize retinochoroidal microvasculature. As glaucoma disease affects retinal ganglion cells in the macula, macular microcirculation is of interest. The purpose of the study was to investigate regional macular vascular characteristics in patients with ocular hypertension (OHT), pre-perimetric primary open-angle glaucoma (pre-POAG) and controls by OCT-A in three microvascular layers.

**Material and methods:**

180 subjects were recruited from the Erlangen Glaucoma Registry, the Department of Ophthalmology, University of Erlangen and residents: 38 OHT, 20 pre-POAG, 122 controls. All subjects received an ophthalmological examination including measurements of retinal nerve fibre layer (RNFL), retinal ganglion cell layer (RGC), inner nuclear layer (INL), and Bruch’s Membrane Opening-Minimum Rim Width (BMO-MRW). Macular vascular characteristics (vessel density, VD, foveal avascular zone, FAZ) were measured by OCT-A (Spectralis OCT II) in superficial vascular plexus (SVP), intermediate capillary plexus (ICP), and deep capillary plexus (DCP).

**Results:**

With age correction of VD data, type 3 tests on fixed effects showed a significant interaction between diagnosis and sectorial VD in SVP (p = 0.0004), ICP (p = 0.0073), and DCP (p = 0.0003). Moreover, a significance in sectorial VD was observed within each layer (p<0.0001) and for the covariate age (p<0.0001). FAZ differed significantly between patients’ groups only in ICP (p = 0.03), not in SVP and DCP. For VD the AUC values of SVP, ICP, and DCP were highest among diagnostic modalities (AUC: 0.88, 95%-CI: 0.75–1.0, p<0.001).

**Conclusion:**

Regional reduced macula VD was observed in all three retinal vascular layers of eyes with OHT and pre-POAG compared to controls, indicating localized microvascular changes as early marker in glaucoma pathogenesis.

## Introduction

Glaucoma is one of the leading causes of visual impairment and blindness worldwide. Yet, its exact pathophysiology is currently unknown. Next to its main risk factor, the elevated intraocular pressure (IOP), i*n vitro* and *in vivo* data support the involvement of a vascular component in glaucoma pathogenesis [1–3]. All conservative, laser and surgical treatments aim to lower the increased IOP to an individual target level in order to win sighted-life time. Yet, despite a regulated IOP, nearly all glaucoma patients show a disease progression. Glaucoma, being a neurodegenerative disease, affects the retinal ganglion cells in the macula region. Thus, potential alterations of its morphometry are of interest in glaucoma research.

The recent introduction of en-face optical coherence tomography angiography (OCT-A) enables non-invasive, fast and high-resolution 3D-images of the retinochoroidal microvasculature (e.g. vessel density, VD). As functional extension of the standard structural OCT, OCT-A uses a motion contrast algorithm to register moving blood cells, causing a temporal change in reflection. Therefore, there is no need for dye injections with contrast agents as used by fluorescein angiography (FA). Retinal microvasculature can be imaged even more precisely by OCT-A than by FA [4]. Previously, OCT-A devices could distinguish between two microvascular layers. The latest version of OCT-A introduced a third retinal vascular layer: superficial vascular plexus (SVP), intermediate capillary plexus (ICP) and deep capillary plexus (DCP), enabling a more fine differentiation of changes in retinal microcirculation [5]. These three layers were shown to correlate well with anatomical structures [6]. In combination with the Erlangen-Angio-Tool (EA-Tool; Erlanger Glaucoma Registry, Erlangen, Germany), retinal microvasculature characteristics can be analyzed in the macula and peripapillary region. As a semi-automated software, the EA-Tool offers a high reliability and reproducibility [7]. All recent studies in early glaucomatous eyes, available up to now in literature, investigate macular vessel density in two retinal layers [8–14]. In addition, early glaucoma is commonly defined as an eye with optic nerve alterations [11, 13] or suspicious-looking optic nerve head [10]. No study is available up to now investigating regional macula vessel density alterations in three retinochoroidal layers in eyes without alterations of the optic disc, yet increased IOP (very early glaucoma suspects).

Thus, the present study aimed to investigate: (I) regional macular vessel density in (II) three different retinal layers by en-face Spectralis II OCT-A in (III) eyes with early glaucomatous alterations (pre-primary open-angle glaucoma, pre-POAG) and eyes without any alterations of the optic nerve heads and increased IOP (very early glaucoma suspects, ocular hypertension, OHT) compared to control eyes. Additionally, ROC analyses were done in comparison to the thickness of retinal nerve fiber layer (RNFL), Bruch’s Membrane Opening Minimum Rim Width (BMO-MRW), thickness of retinal ganglion cell layer (RGC) and inner nuclear layer (INL).

## Material and methods

### Patients

180 patients were recruited from the Erlangen Glaucoma Registry (EGR; ClinicalTrials.gov Identifier: NCT00494923; ISSN 2191-5008, CS-2011) [15], the Department of Ophthalmology, University of Erlangen, and local residents: 58 glaucoma suspects (38 ocular hypertension, OHT, 23 female, 15 male, mean age 66±11 years; 20 pre-perimetric open-angle glaucoma, pre-OAG, 10 female, 10 male, mean age 66±10 years), 122 healthy controls (70 female, 52 male, mean age 34±17 years). All patients and subjects underwent a complete ophthalmological examination, including slit-lamp biomicroscopy, funduscopy, and measurements of IOP. If both eyes met the inclusion criteria, one eye was chosen randomly for analysis. Exclusion criteria were age <18 years, pregnancy, and mental disability. The study has been approved by the local ethics committee of the university of Erlangen-Nuremberg and performed in accordance to the tenets of the Declaration of Helsinki. Informed written consent was achieved from each participant.

Inclusion criteria for control participants, patients with OHT and pre-POAG were:

### Normals

Normal eyes showed no ophthalmological disorder or systemic disease with ophthalmological involvement. Any ophthalmic surgery including refractive surgery was excluded.

### OHT

OHT eyes had normal visual fields in standard white-on-white full-field perimetry (Octopus 500, G1 protocol, Interzeag, Schlieren, Switzerland) according to the following criteria: mean visual field defect (MD) <2.8 dB, <3 adjoining test points with defects p<0.05, and no adjoining test points with defects p<0.01. Optic disc showed no glaucomatous alterations according to the classification of Jonas [16]. IOP was >21 mmHg (repeated twice), measured with Goldmann applanation tonometry. As IOP is affected by central corneal thickness (see review [17], IOP was corrected according to central corneal thickness (CCT) according to Kohlhaas et al. [18] CCT was measured by central ultrasonic pachymetry (Pachymeter SP-100).

### Pre-POAG

Pre-OAG eyes showed an open anterior chamber, normal visual fields (according to criteria for OHT eyes) and a glaucomatous optic disc classified after Jonas [16]. IOP was >21 mmHg (repeated twice), measured with Goldmann applanation tonometry. Intraocular pressure data were corrected according to central corneal thickness according to Kohlhaas et al. [18] CCT was measured by central ultrasonic pachymetry (Pachymeter SP-100).

### Morphometric measurements

Morphometric measurements were done by the Spectralis Optical Coherence Tomography (Spectralis^®^ OCT Version 1.9.10.0, Heidelberg Engineering, Heidelberg, Germany): thickness of retinal nerve fibre layer (RNFL), of the inner, middle, and outer ring of the GMPE (Glaucoma Module Premium Edition), Bruch’s Membrane Opening Minimum Rim Width (BMO-MRW), thickness of retinal ganglion cell layer (RGC) and inner nuclear layer (INL) were measured with Spectral Domain Spectralis II (Heidelberg Engineering, Germany). In order to provide high transverse and axial resolution, a projection artefact removal (PAR) algorithm and TruTrack^®^ eye tracking were used. The scans were checked for artefacts or shadows prior to analysis.

### OCT-A and Erlangen-Angio-Tool

**Fig 1:**
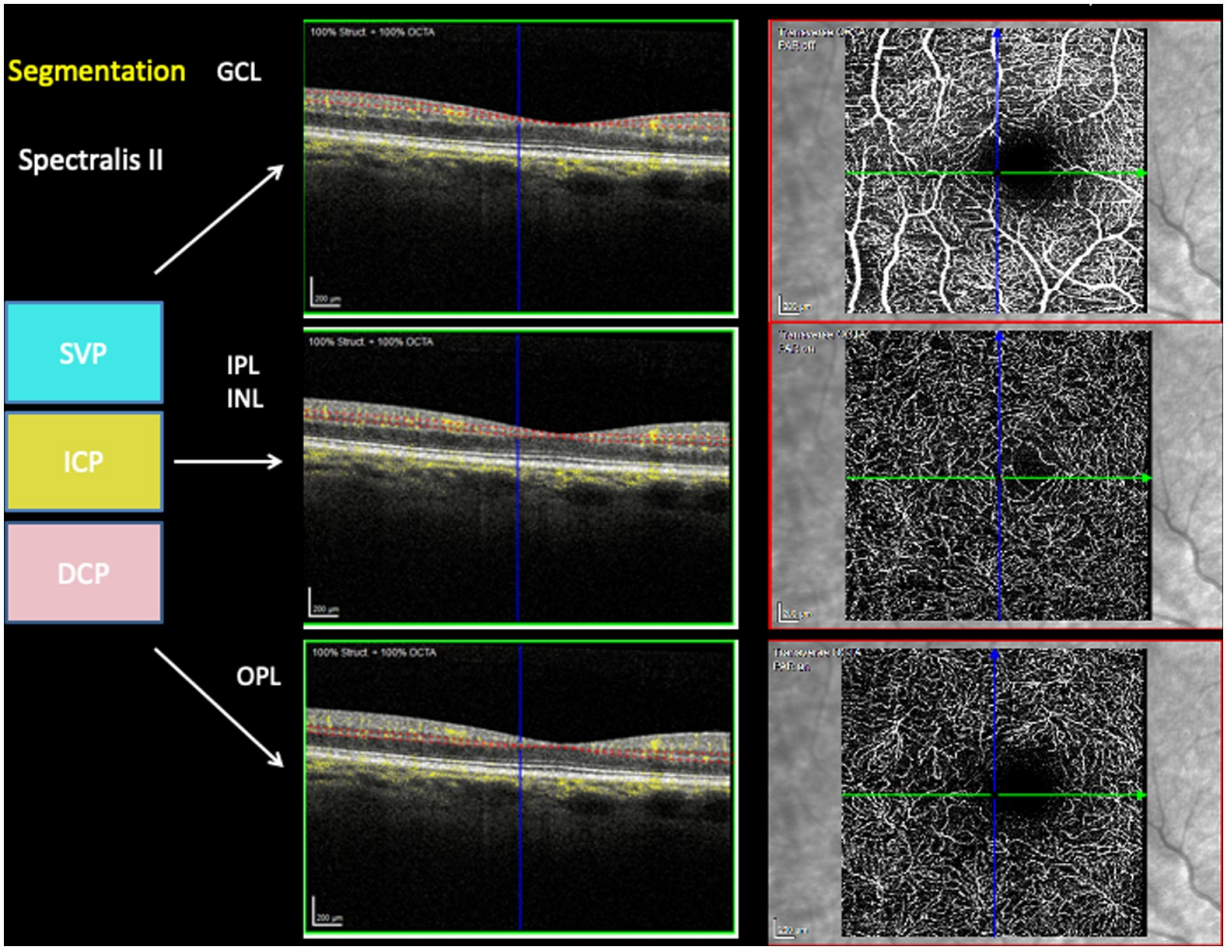
Segmentation of en face OCTA’s superficial vascular plexus (SVP), intermediate capillary plexus (ICP), deep capillary plexus (DCP) in correlation to structure OCT layers’ ganglion cell layer (GCL), inner plexiform layer (IPL) and inner nuclear layer (INL) and outer plexiform layer (OPL).

Macular vascular morphology was scanned with the Heidelberg Spectralis II (Heidelberg, Germany) in three different layers: superficial vascular plexus (SVP, thickness: 80 μm), intermediate capillary plexus (ICP, thickness: 50 μm), and deep capillary plexus (DCP, thickness: 40 μm). The correlation of en face OCT-A layers to the retinal layers in structure OCT are shown in Fig 1. The scans were based on an angle of 15° x 15° and the highest commercially available lateral resolution of 5.7 μm/pixel. Scan size was 2.9 mm x 2.9 mm (total scan size 8.41 mm2) with a diameter of 0.8 mm for the inner ring and 2.9 mm for the outer ring. All scans were analyzed by the EA-Tool (version 1.0), coded in Matlab (The MathWorks, Inc., R2017b), enabling quantification of VD by performing multiple segmentations with a high reliability and reproducibility [7]. The scans were exported into the EA-Tool, and manually checked for correct segmentation and artefacts prior to analysis. Overall and sectorial VD (12 sectors, s1-s12; à 30°) of the macula of each scan were analyzed (total size: 6.10 mm2).

### Statistical analysis

Statistical analysis was done using SAS version 9.3 (SAS Institute Inc., Cary, NC, USA) and pROC package, (RStudio Version 1.0.136 – © 2009–2016 RStudio, Inc.). For the variables BMO-MRW and RGC, INL, and RNFL thickness we applied a covariance analysis (where gender and age were set in the model as corrections factors). The diagnosis was set as a class variable with three levels (control, OHT, pre-OAG). The interactions between diagnosis with gender and age were calculated. In cases in which they were not significant, they were removed from the model. Type III SS test of the multiple comparisons (adjusted with Tukey-Kramer) and 95% CI were reported to evaluate the contribution of the factor. As the experimental design was unbalanced, we estimated the least squares means (LS-means) that correspond to the specified effects for the linear predictor part of the model, and the relative confidence limits. LS-means are closer to reality and represent even more real data, when cofactors occur, compared to means. For the variables vessel density of SVP, ICP, and DCP, we applied a mixed model analysis with repetition measures (gender and age were set as corrections factors). In the model, a random intercept and 12 time measurements as repetitions were set. We specified within the measurements of a Person covariance structure. The interactions between diagnosis and sectors were also calculated together with the p-values of the multiple comparisons (after the Tukey-Kramer adjustment). The 95% CI were reported together with the p-values. As before, the LS-means were calculated. All the models were adjusted with age and gender factors, checking for the significance of such parameters. Those gave us the between-subjects effects. Moreover, we calculated the within-subjects effects using the interactions age*gender. To compare the performance between the two different typology of measurements, we calculated several receiver operator characteristic (ROC) curves and the corresponding area under the curve (AUC) values. As a class variable, we set the diagnosis and we compared the level “control” against level “pre-OAG” for each variable.

## Results

### BMO-MRW

A covariance analysis with gender and age as corrections factors was done. The interactions between diagnosis with gender and age were not significant (p>0.05). Therefore, the model was run again without them. Gender was not observed to have a significant impact on BMO-MRW data (p>0.05). Instead, age showed a moderate significant impact on BMO-MRW (p = 0.047).

**Fig 2:**
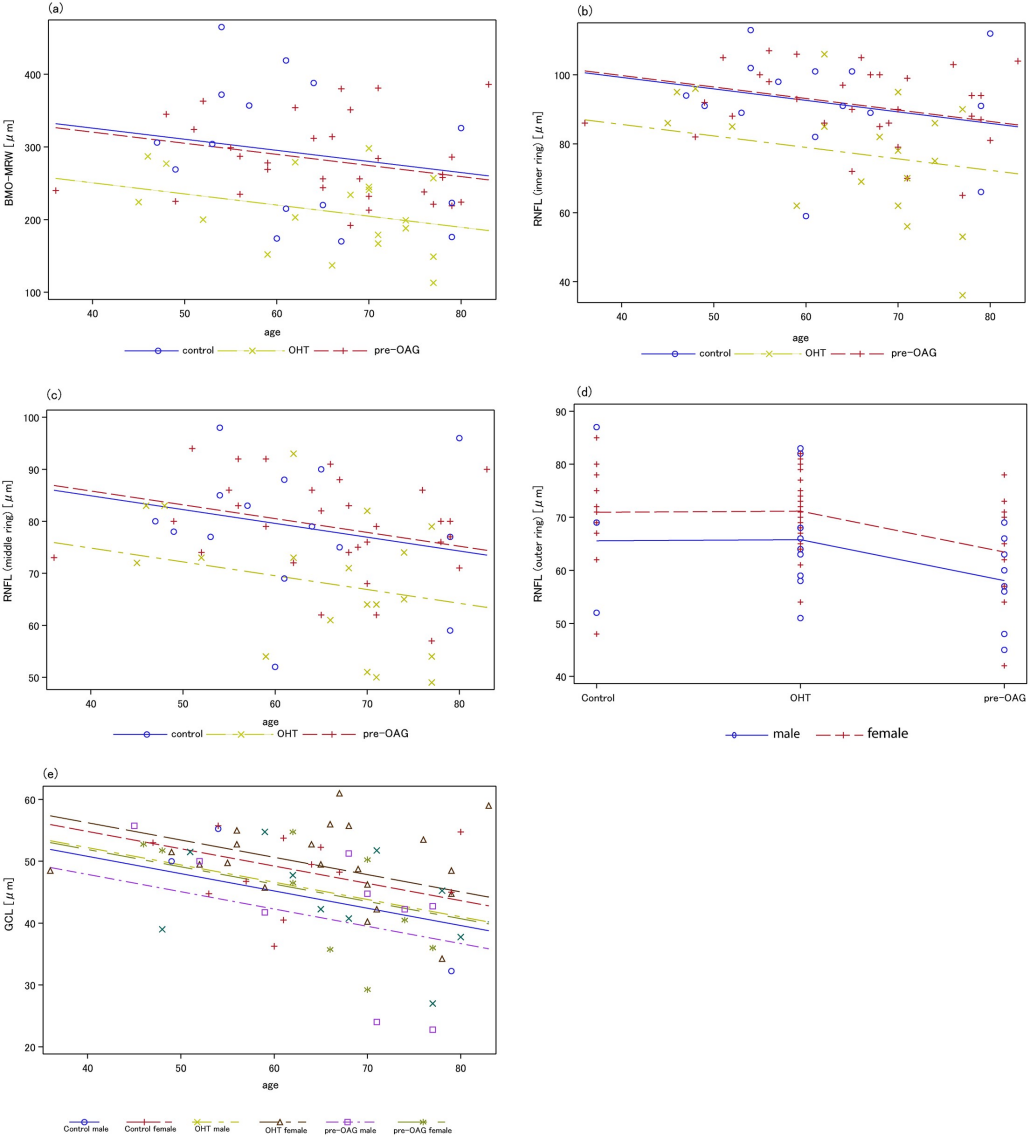
Analysis of covariance for BMO-MRW (a), RNFL (inner, b; middle, c; outer, d), and GCL (e) considering age and patients’ groups. (a) a significant age effect on BMO-MRW was observed (p = 0.047); (b-d) age showed an impact on RFNL for the inner and middle scan; gender was significantly associated with RNFL of the outer scan; (e) a significant decrease of RGC was observed with increasing age for male and female persons (p = 0.0021).

Type III SS test yielded that BMO-MRW was significantly different between patients’ groups and controls (p = 0.0007), respectively. From the analysis of covariance, all the levels of diagnosis were mainly declining across age. BMO-MRW of young controls and patients with OHT was superior compared to young patients with pre-OAG (Fig 2a).

Analysis with multiple comparisons showed that BMO-MRW was significantly different between pre-OAG and controls (CI: 21.19, 129.41; p-value = 0.004) and pre-OAG and OHT (CI: 24.39, 115.14; p-value = 0.001), yet not between OHT and controls (p>0.05).

### RNFL thickness

Thickness of RNFL of the inner and middle ring of the GMPE showed no gender effect (p = 0.067). However, RFNL of the outer ring showed a significant gender effect (p = 0.034, Fig 2d). The interactions were not significant for the inner, middle, and outer circumpapillary scans of RNFL (p>0.05).

Type III SS text yielded a significant effect of diagnosis on RFNL of the inner (p = 0.004), middle (p = 0.008), and outer ring (p = 0.02). An age effect on RFNL was observed for the inner (p = 0.039, Fig 2b) and middle circumpapillary scan (in the border p = 0.053, Fig 2c), yet not for the outer scan (p>0.05).

From the analysis of covariance, it could be observed that all levels of diagnosis were decreasing for males and females with age, with the minimum of the inner scan of RNFL for female young patients with pre-OAG.

Analysis of multiple comparisons showed a significant difference between patients with pre-OAG and controls (inner: CI: 0.44, 23.25; p-value = 0.04; middle (after deletion of gender effect): CI: 0.94, 19.16; p-value = 0.03; outer: CI: 1.30, 14.21; p-value = 0.01) and patients with OHT and pre-OAG (inner: CI: 3.74, 22.65; p-value = 0.004; middle (after deletion of gender effect): CI: 3.29, 18.66; p-value = 0.003).

### RGC thickness

The interactions of diagnosis with gender and age were not significant (p>0.05). Diagnosis of OHT and pre-OAG was not significantly linked to RGC thickness (p>0.05). An age effect (p = 0.0021), yet no gender effect (in the border p = 0.05) on RGC was observed. The analysis of covariance showed that all levels of diagnosis were decreasing with age (for both male and female, Fig 2e). Analysis of multiple comparisons yielded no significances (p>0.05).

### INL thickness

No significant effect of age (p>0.05) and gender (p>0.05) were observed on INL. Analysis of multiple comparisons yielded no significances (p>0.05).

### Vessel density of SVP, ICP, and DCP

Mean and sectorial vessel density can be seen in Table 1 for SVP, ICP, and DCP subdivided for diagnosis. An age effect was observed for mean vessel density in SVP (p<0.0001), ICP (p<0.0001), and DCP (p<0.0001).

**Table 1:** Mean (a) and sectorial (1–12, b) vessel density in SVP, ICP, and DCP for controls, patients with OHT and pre-OAG.

a)													
**Effect**	**Diagnosis**	**Least Squares Means SVP**				**Least Squares Means ICP**				**Least Squares Means DCP**			
		**Estimate**	**Standard error**	**Lower**	**Upper**	**Estimate**	**Standard error**	**Lower**	**Upper**	**Estimate**	**Standard error**	**Lower**	**Upper**
**Diagnosis**	**control**	31.31	0.32	30.68	31.95	22.22	0.27	21.69	22.74	24.31	0.32	23.68	24.95
	**OHT**	31.09	0.61	29.9	32.27	21.62	0.5	20.63	22.6	23.04	0.6	21.86	24.22
	**pre-OAG**	28.41	0.77	26.9	29.93	21.03	0.64	19.78	22.28	23.14	0.77	21.64	24.64
**b)**													
**Diagnosis**	**Sector**	**Least Squares Means SVP**				**Least Squares Means ICP**				**Least Squares Means DCP**			
		**Estimate**	**Standard error**	**Lower**	**Upper**	**Estimate**	**Standard error**	**Lower**	**Upper**	**Estimate**	**Standard error**	**Lower**	**Upper**
**controls**	1	32.05	0.39	31.29	32.8	21.59	0.33	20.94	22.23	24.55	0.4	23.76	25.34
	2	31.51	0.39	30.75	32.27	21.71	0.33	21.06	22.35	24.52	0.4	23.73	25.31
	3	30.81	0.39	30.06	31.57	23.5	0.33	22.85	24.14	25.54	0.4	24.75	26.33
	4	31.37	0.39	30.61	32.12	23.84	0.33	23.19	24.48	25.59	0.4	24.8	26.38
	5	31.68	0.39	30.92	32.44	21.94	0.33	21.29	22.58	24.13	0.4	23.34	24.92
	6	31.94	0.39	31.18	32.69	21.46	0.33	20.82	22.1	23.82	0.4	23.03	24.61
	7	32.17	0.39	31.41	32.93	21.47	0.33	20.82	22.11	23.51	0.4	22.71	24.3
	8	31.54	0.39	30.78	32.3	21.71	0.33	21.07	22.36	23.36	0.4	22.57	24.15
	9	30.04	0.39	29.28	30.8	23.25	0.33	22.61	23.89	24.27	0.4	23.48	25.06
	10	29.57	0.39	28.81	30.32	23.01	0.33	22.36	23.65	24.19	0.4	23.4	24.99
	11	31	0.39	30.24	31.76	21.65	0.33	21	22.29	24.03	0.4	23.24	24.82
	12	32.07	0.39	31.31	32.83	21.49	0.33	20.85	22.14	24.28	0.4	23.49	25.08
**OHT**	1	31.78	0.71	30.38	33.18	20.73	0.61	19.55	21.92	23.06	0.74	21.61	24.51
	2	31.4	0.71	30	32.8	21.24	0.61	20.05	22.43	23.31	0.74	21.86	24.76
	3	31.2	0.71	29.8	32.6	22.84	0.61	21.65	24.03	24.84	0.74	23.39	26.29
	4	32.07	0.71	30.67	33.47	23.33	0.61	22.14	24.52	25.26	0.74	23.81	26.71
	5	31.3	0.71	29.89	32.7	21.26	0.61	20.07	22.45	22.5	0.74	21.05	23.95
	6	31.42	0.71	30.02	32.83	20.37	0.61	19.18	21.56	21.6	0.74	20.15	23.05
	7	31.98	0.71	30.58	33.38	20.76	0.61	19.57	21.95	22.08	0.74	20.63	23.53
	8	31.19	0.71	29.79	32.59	21.35	0.61	20.16	22.54	22.67	0.74	21.22	24.12
	9	29.75	0.71	28.35	31.15	23.16	0.61	21.97	24.35	23.47	0.74	22.02	24.92
	10	29.35	0.71	27.95	30.75	22.6	0.61	21.41	23.79	23.08	0.74	21.63	24.53
	11	30.23	0.71	28.83	31.64	20.79	0.61	19.6	21.98	21.97	0.74	20.52	23.42
	12	31.36	0.71	29.96	32.76	20.95	0.61	19.76	22.14	22.61	0.74	21.16	24.06
**pre-OAG**	1	29.58	0.93	27.75	31.41	19.98	0.79	18.43	21.54	23.25	0.97	21.35	25.15
	2	29.46	0.93	27.63	31.29	21.31	0.79	19.76	22.87	24.05	0.97	22.16	25.95
	3	30.02	0.93	28.19	31.85	23.78	0.79	22.23	25.34	26.68	0.97	24.78	28.58
	4	29.6	0.93	27.78	31.43	24.19	0.79	22.64	25.75	26.99	0.97	25.09	28.88
	5	28.38	0.93	26.55	30.21	20.68	0.79	19.12	22.23	23.05	0.97	21.15	24.95
	6	27.49	0.93	25.67	29.32	18.72	0.79	17.17	20.28	21.7	0.97	19.8	23.6
	7	28.09	0.93	26.26	29.92	19.16	0.79	17.6	20.71	21.07	0.97	19.18	22.97
	8	26.84	0.93	25.01	28.67	19.4	0.79	17.84	20.95	20.61	0.97	18.71	22.51
	9	26.36	0.93	24.53	28.18	22.46	0.79	20.91	24.02	23.24	0.97	21.34	25.13
	10	26.63	0.93	24.8	28.46	22.16	0.79	20.6	23.71	22.63	0.97	20.73	24.52
	11	28.36	0.93	26.53	30.18	20.38	0.79	18.82	21.93	21.99	0.97	20.09	23.89
	12	30.13	0.93	28.31	31.96	20.15	0.79	18.6	21.71	22.44	0.97	20.54	24.34

Gender did show an effect only on LS-mean VD of the DCP (p = 0.0338), yet not on SVP and ICP (p>0.05), respectively. The data of the type III tests of fixed effects were presented in Table 2.

**Table 2:** Type III tests of fixed effects for SVP (a), ICP (b), and DCP (c): Diagnosis, OCT-A sector, age and the interaction diagnosis with sector is presented.

(a)				
**Type 3 Tests of Fixed Effects**				
Effect	Num DF	Den DF	F Value	Pr > F
Diagnosis	2	1947	6.16	0.0021
OCT-A sector	11	1947	14.11	<.0001
Age	1	1947	43.26	<.0001
Diagnosis*Sector	22	1947	2.37	0.0004
(b)				
**Type 3 Tests of Fixed Effects**				
Effect	Num DF	Den DF	F Value	Pr > F
Diagnosis	2	1947	1.31	NS
OCT-A sector	11	1947	32.84	<.0001
Age	1	1947	45.52	<.0001
Diagnosis*Sector	22	1947	1.89	0.007
(c)				
**Type 3 Tests of Fixed Effects**				
Effect	Num DF	Den DF	F Value	Pr > F
Diagnosis	2	1947	1.62	NS
OCT-A sector	11	1947	20.92	<.0001
Gender	1	1947	4.51	0.034
Age	1	1947	42.22	<.0001
Diagnosis*Sector	22	1947	2.40	0.0003

With age correction of VD data, mean VD of SVP was significantly associated with diagnosis (p = 0.0021). This effect was not observed for mean VD of ICP (p = 0.2690) and DCP (p = 0.1975). Considering a potential variation of VD due to their localization, type III tests of fixed effects offered a significant association of the sector (s1-s12) on VD in all the layers (SVP, ICP and DCP, p<0.0001). Analysis of the interaction between diagnosis and sector yielded a significance for SVP (p = 0.0004), ICP (p = 0.0073), and DCP (p = 0.0003).

**Fig 3:**
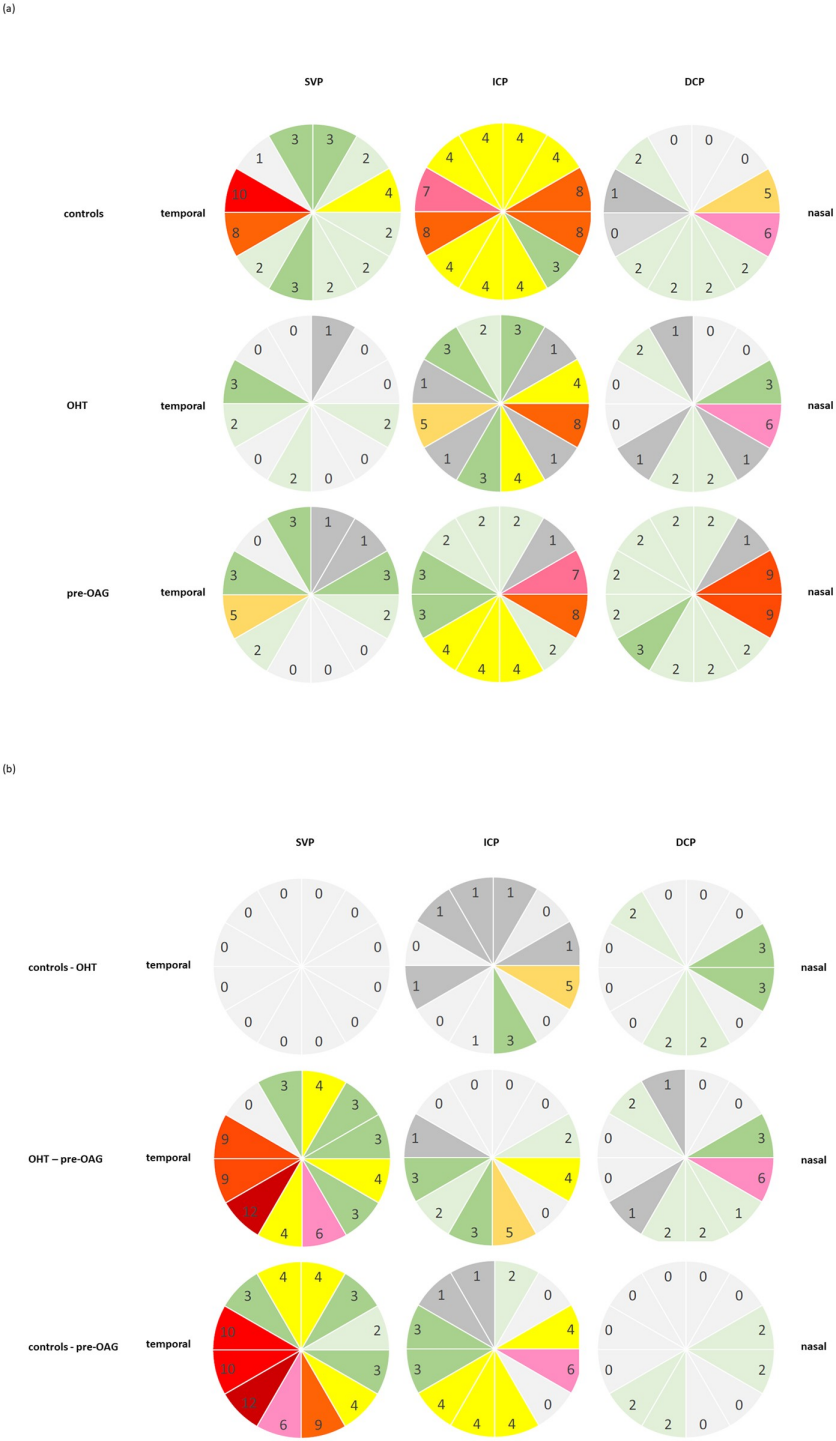
Qualitative analysis of the number of significant interactions between vessel density of each sector (s1-s12) of macula OCT-A in SVP, ICP, and DCP by color coding (red, n = 8–12; pink, n = 6–7; orange, n = 5; yellow, n = 4; green, n = 2–3; grey, n = 0–1) in controls, OHT, and pre-OAG eyes, respectively (a) and between the groups (b) with age correction of the data: Notice the temporal emphasis in SVP and the nasal emphasis in DCP.

Color coded numbers of all significant interactions (Pr > |t|; p<0.002) between each sector can be seen in Fig 3 for each vascular layer, respectively (number of significant interactions: red, n = 8–12; pink, n = 6–7; orange, n = 5; yellow, n = 4; green, n = 2–3; grey, n = 0–1). The localization of significant microvascular changes was even pronounced in temporal regions in SVP, and in nasal regions in DCP of control and glaucoma eyes. The data of all significant interactions are presented in S1 Table.

### FAZ area characteristics of SVP, ICP, and DCP

A general linear model analysis (as for the first part) was done.

**Fig 4:**
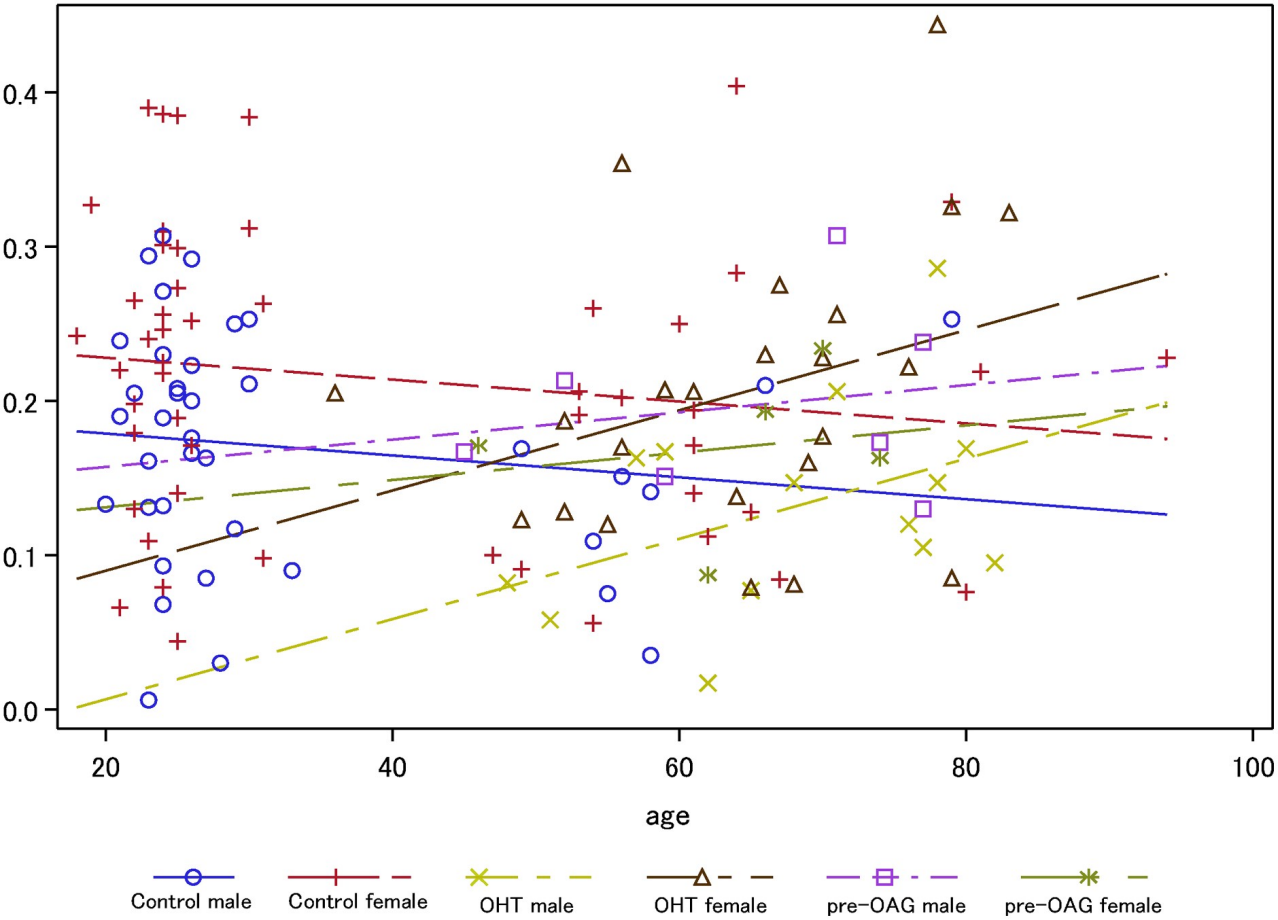
Analysis of covariance for FAZ of ICP across age subdivided for gender and diagnosis (OHT, pre-OAG, controls).

FAZ area was significantly different between patients with OHT, pre-OAG and controls (p = 0.03) in ICP, yet not in SVP and DCP (p>0.05), respectively. Furthermore, a gender effect on FAZ was observed in DCP (p = 0.0079), yet not in SVP (p>0.05) and ICP (p>0.05). The interaction of diagnosis with age yielded a significant association with FAZ only in ICP (p = 0.0456): FAZ is reduced with increasing age in control subjects. On the contrary, FAZ of ICP increased with age in patients with OHT and pre-OAG. It seemed that the increase of FAZ is even more prominent in patients with OHT than pre-OAG (Fig 4).

### ROC of vessel density characteristics and morphometric parameters

**Fig 5:**
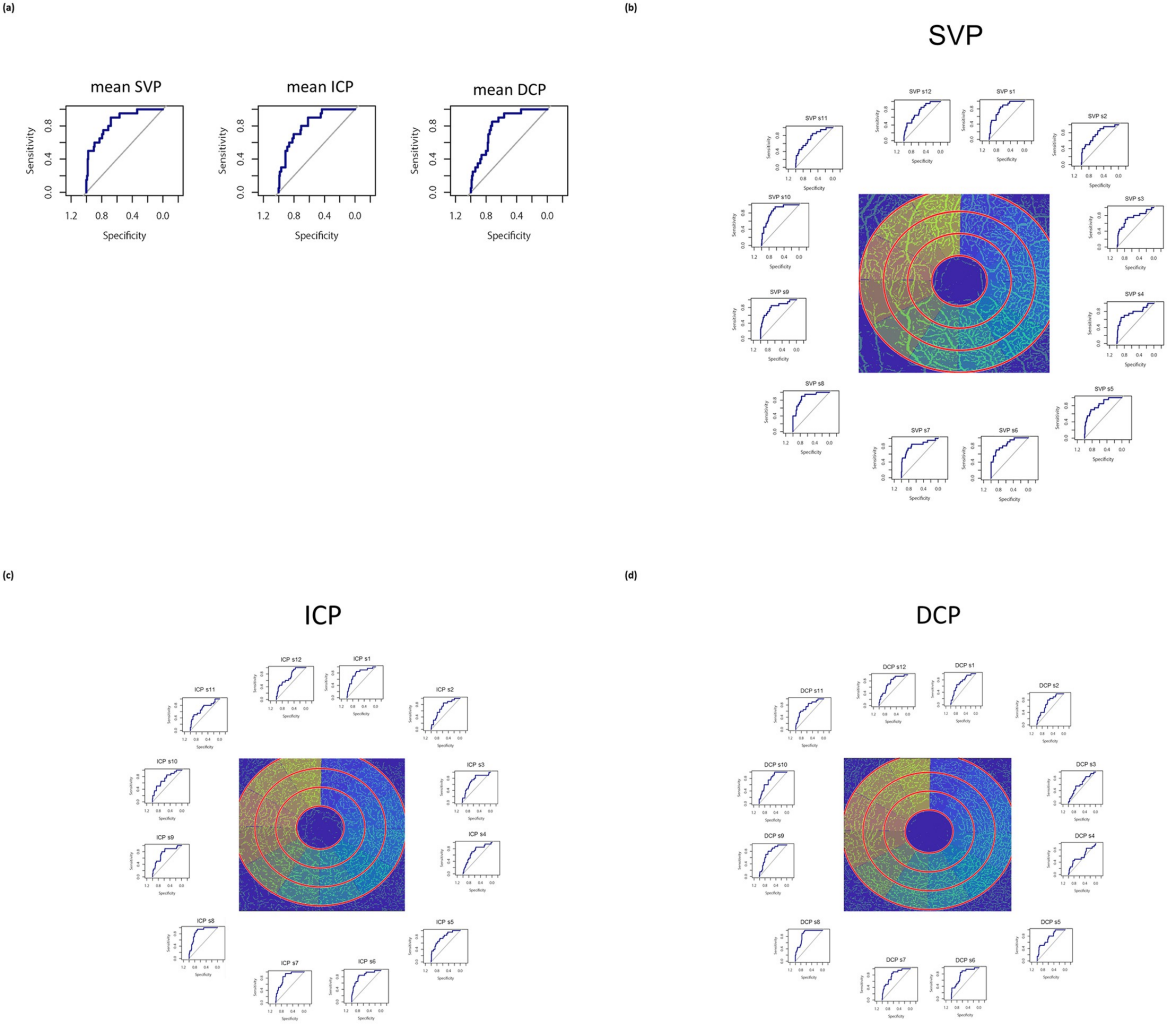
Receiver operating curves (ROC) of mean (a) and sectorial (b-d) vessel density in SVP, ICP, and DCP for differentiation between patients’ group and controls.

ROC analyses were done for mean and sectorial VD of SVP, ICP, and DCP (Fig 5). Additionally, ROC curves of RNFL (inner, middle, outer ring), BMO-MRW, RGC, and INL were calculated (see S1 Fig).

Area under the curve (AUC) of mean VD was 0.8525 (SVP), 0.8234 (ICP), and 0.8098 (DCP). Sectorial AUC ranged between 0.7488–0.877 (SCP), 0.7068–0.8619 (ICP), and 0.6004–0.8568 (DCP, Table 3a). Additionally, mean AUC was 0.7439 (BMO-MRW), 0.7561 (RNFL, inner ring), 0.7421 (RNFL, middle ring), 0.7246 (RNFL, outer ring), 0.65 (GCL), and 0.5278 (INL, Table 3b).

**Table 3:** Area under the curve (AUC) for sectorial macula VD in SVP, ICP, and DCP (a) and glaucoma morphometric parameters (BMO-MRW, RNF (inner, middle, outer ring), GCL, INL, (b).

a)			
**sector**	**SVP**	**AUC ICP**	**DCP**
**1**	0.8238	0.7893	0.7338
**2**	0.7695	0.7244	0.7113
**3**	0.7488	0.7203	0.6266
**4**	0.7734	0.7203	0.6004
**5**	0.8303	0.7871	0.7525
**6**	0.8451	0.8395	0.7799
**7**	0.8234	0.8393	0.8004
**8**	0.877	0.8619	0.8568
**9**	0.8201	0.759	0.7734
**10**	0.851	0.7301	0.7746
**11**	0.7564	0.7068	0.7561
**12**	0.7559	0.7365	0.7475
b)			
			**AUC**
**BMO-MRW**		global	0.7439
		nasal superior	0.7175
		nasal	0.6684
		nasal inferior	
		temporal inferior	0.8175
		temporal	0.7649
		temporal superior	0.7333
**RNLF inner ring**		global	0.7561
		nasal superior	0.7877
		nasal	0.6053
		nasal inferior	0.6368
		temporal inferior	0.6947
		temporal	0.7088
		temporal superior	0.6439
**RNLF middle ring**		global	0.7421
		nasal superior	0.8140
		nasal	0.5965
		nasal inferior	0.6456
		temporal inferior	0.6737
		temporal	0.6561
		temporal superior	0.6596
**RNLF outer ring**		global	0.7246
		nasal superior	0.8228
		nasal	0.5930
		nasal inferior	0.6211
		temporal inferior	0.6754
		temporal	0.6298
		temporal superior	0.6789
**GCL**		global	0.6500
		superior	0.6185
		nasal	0.6259
		inferior	0.6667
		temporal	0.6444
**INL**		global	0.5278
		superior	0.6074
		nasal	0.5778
		inferior	0.5370
		temporal	0.4981

## Discussion

Glaucoma is one of the leading causes of blindness worldwide. An increase to over 100 million affected people is estimated in 2040 [19]. Although several risk factors have been established up to now, the exact pathophysiology is still unknown. Involvement of an impaired blood flow in the etiopathogenesis is assumed and substantiated by several studies [1, 2]. With the introduction of OCT-A it is possible to measure the retinochoroidal microvasculature non-invasively within specific layers of the retina as well as obtaining reproducible quantitative measurements [7, 20]. Recent studies were done by different OCT-A devices, commonly differentiating the microvasculature in two layers, yet only Spectralis OCT-A offers a subdivision into three microvascular layers in macula scans. These three vascular plexuses corresponded well with anatomical structures [6]. In addition, a structure–function association was observed as loss of OCT-A macula VD was associated with defects in central 10–2 visual fields [21]. As recent OCT-A findings were observed in the macular region, subdivided into two layers, of patients with manifest and/or early glaucoma or with suspicious-looking optic nerve head, the purpose of this study was to investigate regional retinochoroidal microcirculation in the macula region of eyes with OHT and pre-POAG by en-face OCT-A in three microvascular layers compared to healthy eyes. An age effect was observed of LS-means VD in SVP, ICP, and DCP. With age correction of vessel density data, LS-mean VD differed significantly between the three microvascular layers and diagnosis, respectively. ROC analysis showed that AUC of VD was even superior compared to BMO-MRW, RNFL, GCL, and INL.

OCT-A enables scanning of high-resolution images with consecutive analysis of retinochoroidal microcirculation in different vascular layers. Activation PAR (projection artefact removal) diminishes scanning artefacts due to e.g. eye movements. As an extension of the standard structural OCT, OCT-A uses a motion contrast algorithm to detect moving blood cells. The temporal change in reflection caused by blood cells allows a non-invasive examination of retinal microcirculation in the macula and peripapillary region. Quantitative analysis of microvasculature characteristics can be done by the EA-tool. This semi-automated software presents high reliability and reproducibility [7]. Macula vessel density was observed to be reduced with increasing age in all three vascular layers of the present cohort, confirming previous findings [22–24]. Contrary to data in literature [24] investigating FAZ areas of healthy eyes, the data of FAZ area yielded an age effect in ICP. Additionally, a gender effect was observed for VD and FAZ area of DCP, but not of ICP and SVP. On the contrary, subdivision into only two vascular layers (superficial vascular complex (SVC) and deep vascular complex (DVC) showed no gender effect at all [24, 25]. This observation of different ‘gender’ effects could be due to the very fine alterations between VD and FAZ characteristics of men and women, thus only a differentiation into 3 vascular layers may be able to detect them.

With age correction of the data, macula mean LS-VD was significantly reduced in all three vascular layers (SVP, ICP, DCP) in eyes with OHT and pre-OAG compared to healthy controls. Overall LS-mean VD differed significantly between OHT and pre-OAG patients in SVP, yet not in ICP and DCP. These data confirm with recently published studies. Yet, these studies were done with a different OCT-A device (Avanti AngioVue, Optovue Inc., USA) with quantitative analysis of two different vascular layers (superficial, SL; deep, DL retinal vascular plexus). Scan size differed as well (3 mm x 3 mm; 6 mm x 6 mm) [10, 26, 27]. Macula VD of SL was significantly associated with RNFL and GCL loss, yet, macula VD of DL was not analyzed in this study [10]. Further studies showed a significant reduction of VD of SL and DL means in glaucomatous eyes [26, 27]. Considering that localized defects of VD might be detectable even earlier in glaucoma pathogenesis than overall mean VD defects, a sectorial analysis of VD of the macula was done after age correction of the data. Localized VD variations were obvious in all three vascular layers of eyes with OHT and pre-POAG with a typical location temporal inferior with the present age corrected VD data. This argues for a distinct analysis of VD in different macula sectors in different vascular layers, respectively.

ROC analysis yielded even higher AUC of VD (SVP, ICP, and DCP) compared to AUC of BMO-MRW, RNFL, INL, and GCL when comparing glaucoma suspects to control eyes. As BMO-MRW was introduced as an early glaucoma marker (AUC = 0.95 [28]; AUC = 0.96 [29]), analysis of macula microvasculature characteristics by OCT-A seemed to provide a novel option in glaucoma diagnosis. These alterations of the microcirculation of the macula seemed to occur even earlier than neuronal alterations presented by BMO-MRW.

Our study is not without limitations. Study participants were white Caucasians, thus we represent a European study cohort. As ethnicity [24] was seen to influence retinochoroidal microcirculation, the present data should not be seen as overall data.

The data of the present study might give a further hint at answering the question of “what was first”—an impaired microcirculation or the neuronal damage? As patients with OHT showed an impaired retinochoroidal microcirculation, yet no glaucomatous alterations of the optic disc, we propose that the altered microcirculation is one of the factors prior to neuronal damage and potentially inducing it, considering neurovascular coupling.

Capillary blood flow can be regulated locally by the endothelium, forming the inner wall as well as by the pericytes from exterior. An impaired microcirculation is supposed to be associated with endothelial dysfunction and oxidative stress (e.g. observed for cardiovascular disorders and diabetes mellitus type 2) [30]. Oxidants, antioxidants (via nitrogen species, reactive oxygen) and vasodilator agents (e.g. nitric oxide) act and interact in this complex vascular regulation (see review) [31]. The blood-retina barrier (BRB) is arranged by tight junctions between the retinal pigment epithelium (RPE, outer BRB) and the endothelial cells of retinal capillaries (inner BRB). Oxidative stress was seen to induce alterations of the molecular structure of endothelial tight junctions [32], with consecutive break-down of the inner BRB. *In vitro* experiments showed that endothelial cells were much more vulnerable to oxidative stress than to acute high pressure peaks [33]. In addition, hyperhomocysteinemia, being a further risk factor in glaucoma pathogenesis, is able to induce endothelial dysfunction via oxidative stress [34]. Endothelial dysfunction is defined by impaired vasodilatation, which is caused by a reduced level of nitric oxide (NO) of the endothelium. NO is a potent vasodilator, produced by type III isoform of nitric oxide synthase (eNOS) of the endothelial cells (EC). Thus, the impaired bioavailability of NO could be due to a reduced expression [35] or decreased activity (e.g. missing cofactors [36, 37]) of eNOS or even accelerated degradation of NO [38]. Reduced levels of NO were measured in the aqueous humour of patients with glaucoma compared to healthy controls [39]. Inhibition of eNOS was observed to reduce blood flow in the choroid and optic nerve head in animals and humans [40–42]. To confirm to this observation, ONH blood flow was increased by NO donors [43]. NO can be supported by nutrition (dietary nitrates, dark green leafy vegetables). The Nurses’ Health Study and Health Professionals Follow-up Study suggested that higher nutritive uptake of nitrate was associated with a lower risk for glaucoma [44]. Even glaucoma worsening seemed to be reduced by the intake of nitric oxide (NO) (see review [45]).

Endothelial cells share their basement membrane with overlying pericytes. These contractile cells form the outer surface of the capillaries [46] The interaction between both cell types enables blood flow regulation [47]. A crosstalk of 1:1 between EC and pericytes is the basis for a fine adjustment between the inner and outer capillary wall (see review [48]). Pericytes are involved in microvascular blood flow control and neurovascular coupling (e.g. in CNS) [49, 50]. *In vitro* data showed that pericytes contract after application of norepinephrine and relax after vasoactive intestinal peptide (VIP) [51]. Thus, all factors influencing pericytes (e.g. autonomic nervous system, antibodies against adrenergic receptors) [52] and mechanical environmental alterations (e.g. shear or tensile forces [53]) may be able to influence capillary microcirculation.

## Conclusion

An overall and regional reduction of macula VD in the sectorial analysis of SVP, ICP, and DCP in eyes with pre-POAG and especially OHT argue for an influence of retinochoroidal microcirculation very early in glaucoma pathogenesis. The present data argue for a critical role of ocular blood flow, which is present even earlier than neuronal damage of the optic disc.

## Supporting information

S1 Fig Receiver operating curves (ROC) for BMO-MRW (a), RNFL (inner, b; middle, c; outer ring, d), RGC (e), and INL (f) for differentiation between patients’ group and controls. (TIF)

S1 Table Differences between sectorial LS-means for the mixed model analysis of SVP (a), ICP (b), and DCP (c) subgrouped by diagnosis (0 –control, 1 –OHT, 2 –pre-OAG). All the possible multiple comparisons are presented together with the p-values, lower and upper values and the correspondent adjusted values.
(DOCX)
