# Supplementary material for: OCT-angiography: Regional reduced macula microcirculation in ocular hypertensive and pre-perimetric glaucoma patients
Source: PLoS One. 2021 Feb 11;16(2):e0246469. doi: 10.1371/journal.pone.0246469 (PMC7877568; doi:10.1371/journal.pone.0246469)
Supplement: S1 Table — All the possible multiple comparisons are presented together with the p-values, lower and upper values and the correspondent adjusted values. (DOCX) [file pone.0246469.s002.docx]

| *a)* | | | | | | | | | | | | | | | | |
| --- | --- | --- | --- | --- | --- | --- | --- | --- | --- | --- | --- | --- | --- | --- | --- | --- |
| ***SVP*** | | | | | | | | | | | | | | | | |
| ***Differences of Least Squares Means*** | | | | | | | | | | | | | | | | |
| ***Effect*** | ***Sector*** | ***diagnosis*** | ***Sector*** | ***diagnosis*** | ***Estimate*** | ***Standard*** | ***DF*** | ***t Value*** | ***Pr > \|t\|*** | ***Adjustment*** | ***Adj P*** | ***Alpha*** | ***Lower*** | ***Upper*** | ***Adj Lower*** | ***Adj Upper*** |
|  |  |  |  |  |  | ***Error*** |  |  |  |  |  |  |  |  |  |  |
| ***sector*** | ***6*** |  | ***9*** |  | *1.57* | *0.33* | *1947* | *4.69* | *<.0001* | *Tukey-Kramer* | *0.0002* | *0.05* | *0.91* | *2.22* | *0.47* | *2.66* |
| ***sector*** | ***10*** |  | ***11*** |  | *-1.35* | *0.33* | *1947* | *-4.03* | *<.0001* | *Tukey-Kramer* | *0.0033* | *0.05* | *-2.00* | *-0.69* | *-2.44* | *-0.25* |
| ***sector*** | ***10*** |  | ***8*** |  | *-1.34* | *0.33* | *1947* | *-4.01* | *<.0001* | *Tukey-Kramer* | *0.0036* | *0.05* | *-2.00* | *-0.69* | *-2.44* | *-0.25* |
| ***sector*** | ***12*** |  | ***8*** |  | *1.33* | *0.33* | *1947* | *3.98* | *<.0001* | *Tukey-Kramer* | *0.0041* | *0.05* | *0.67* | *1.99* | *0.24* | *2.42* |
| ***sector*** | ***11*** |  | ***12*** |  | *-1.32* | *0.33* | *1947* | *-3.96* | *<.0001* | *Tukey-Kramer* | *0.0044* | *0.05* | *-1.98* | *-0.67* | *-2.42* | *-0.23* |
| ***sector*** | ***1*** |  | ***8*** |  | *1.28* | *0.33* | *1947* | *3.82* | *0.0001* | *Tukey-Kramer* | *0.0076* | *0.05* | *0.62* | *1.93* | *0.18* | *2.37* |
| ***sector*** | ***1*** |  | ***11*** |  | *1.27* | *0.33* | *1947* | *3.8* | *0.0001* | *Tukey-Kramer* | *0.0082* | *0.05* | *0.62* | *1.93* | *0.18* | *2.37* |
| ***sector*** | ***4*** |  | ***8*** |  | *1.16* | *0.33* | *1947* | *3.46* | *0.0005* | *Tukey-Kramer* | *0.0271* | *0.05* | *0.50* | *1.81* | *0.06* | *2.25* |
| ***sector*** | ***11*** |  | ***4*** |  | *-1.15* | *0.33* | *1947* | *-3.44* | *0.0006* | *Tukey-Kramer* | *0.029* | *0.05* | *-1.81* | *-0.50* | *-2.25* | *-0.06* |
| ***sector*** | ***11*** |  | ***9*** |  | *1.15* | *0.33* | *1947* | *3.43* | *0.0006* | *Tukey-Kramer* | *0.0304* | *0.05* | *0.49* | *1.80* | *0.05* | *2.24* |
| ***sector*** | ***8*** |  | ***9*** |  | *1.14* | *0.33* | *1947* | *3.41* | *0.0007* | *Tukey-Kramer* | *0.0326* | *0.05* | *0.48* | *1.80* | *0.05* | *2.23* |
| ***sector*** | ***1*** |  | ***10*** |  | *2.62* | *0.33* | *1947* | *7.83* | *<.0001* | *Tukey-Kramer* | *<.0001* | *0.05* | *1.96* | *3.27* | *1.53* | *3.71* |
| ***sector*** | ***1*** |  | ***9*** |  | *2.42* | *0.33* | *1947* | *7.23* | *<.0001* | *Tukey-Kramer* | *<.0001* | *0.05* | *1.76* | *3.07* | *1.32* | *3.51* |
| ***sector*** | ***10*** |  | ***12*** |  | *-2.67* | *0.33* | *1947* | *-7.99* | *<.0001* | *Tukey-Kramer* | *<.0001* | *0.05* | *-3.33* | *-2.02* | *-3.77* | *-1.58* |
| ***sector*** | ***10*** |  | ***2*** |  | *-2.28* | *0.33* | *1947* | *-6.81* | *<.0001* | *Tukey-Kramer* | *<.0001* | *0.05* | *-2.93* | *-1.62* | *-3.37* | *-1.18* |
| ***sector*** | ***10*** |  | ***3*** |  | *-2.16* | *0.33* | *1947* | *-6.47* | *<.0001* | *Tukey-Kramer* | *<.0001* | *0.05* | *-2.82* | *-1.51* | *-3.26* | *-1.07* |
| ***sector*** | ***10*** |  | ***4*** |  | *-2.50* | *0.33* | *1947* | *-7.48* | *<.0001* | *Tukey-Kramer* | *<.0001* | *0.05* | *-3.15* | *-1.84* | *-3.59* | *-1.41* |
| ***sector*** | ***10*** |  | ***5*** |  | *-1.94* | *0.33* | *1947* | *-5.79* | *<.0001* | *Tukey-Kramer* | *<.0001* | *0.05* | *-2.59* | *-1.28* | *-3.03* | *-0.84* |
| ***sector*** | ***10*** |  | ***6*** |  | *-1.77* | *0.33* | *1947* | *-5.29* | *<.0001* | *Tukey-Kramer* | *<.0001* | *0.05* | *-2.43* | *-1.11* | *-2.86* | *-0.68* |
| ***sector*** | ***10*** |  | ***7*** |  | *-2.23* | *0.33* | *1947* | *-6.68* | *<.0001* | *Tukey-Kramer* | *<.0001* | *0.05* | *-2.89* | *-1.58* | *-3.33* | *-1.14* |
| ***sector*** | ***12*** |  | ***9*** |  | *2.47* | *0.33* | *1947* | *7.39* | *<.0001* | *Tukey-Kramer* | *<.0001* | *0.05* | *1.81* | *3.13* | *1.38* | *3.56* |
| ***sector*** | ***2*** |  | ***9*** |  | *2.07* | *0.33* | *1947* | *6.21* | *<.0001* | *Tukey-Kramer* | *<.0001* | *0.05* | *1.42* | *2.73* | *0.98* | *3.17* |
| ***sector*** | ***3*** |  | ***9*** |  | *1.96* | *0.33* | *1947* | *5.87* | *<.0001* | *Tukey-Kramer* | *<.0001* | *0.05* | *1.31* | *2.62* | *0.87* | *3.06* |
| ***sector*** | ***4*** |  | ***9*** |  | *2.30* | *0.33* | *1947* | *6.87* | *<.0001* | *Tukey-Kramer* | *<.0001* | *0.05* | *1.64* | *2.95* | *1.20* | *3.39* |
| ***sector*** | ***5*** |  | ***9*** |  | *1.74* | *0.33* | *1947* | *5.19* | *<.0001* | *Tukey-Kramer* | *<.0001* | *0.05* | *1.08* | *2.39* | *0.64* | *2.83* |
| ***sector*** | ***7*** |  | ***9*** |  | *2.03* | *0.33* | *1947* | *6.08* | *<.0001* | *Tukey-Kramer* | *<.0001* | *0.05* | *1.38* | *2.69* | *0.94* | *3.13* |
| ***diagnosis*sector*** | ***5*** | ***0*** | ***9*** | ***0*** | *1.64* | *0.31* | *1947* | *5.24* | *<.0001* | *Tukey-Kramer* | *0.0001* | *0.05* | *1.02* | *2.25* | *0.44* | *2.84* |
| ***diagnosis*sector*** | ***7*** | ***0*** | ***10*** | ***2*** | *5.54* | *1.06* | *1947* | *5.24* | *<.0001* | *Tukey-Kramer* | *0.0001* | *0.05* | *3.46* | *7.61* | *1.47* | *9.60* |
| ***diagnosis*sector*** | ***7*** | ***1*** | ***9*** | ***2*** | *5.63* | *1.08* | *1947* | *5.21* | *<.0001* | *Tukey-Kramer* | *0.0001* | *0.05* | *3.51* | *7.74* | *1.48* | *9.77* |
| ***diagnosis*sector*** | ***1*** | ***0*** | ***10*** | ***2*** | *5.41* | *1.06* | *1947* | *5.12* | *<.0001* | *Tukey-Kramer* | *0.0002* | *0.05* | *3.34* | *7.49* | *1.35* | *9.48* |
| ***diagnosis*sector*** | ***12*** | ***0*** | ***10*** | ***2*** | *5.44* | *1.06* | *1947* | *5.14* | *<.0001* | *Tukey-Kramer* | *0.0002* | *0.05* | *3.36* | *7.51* | *1.37* | *9.50* |
| ***diagnosis*sector*** | ***5*** | ***0*** | ***9*** | ***2*** | *5.32* | *1.06* | *1947* | *5.03* | *<.0001* | *Tukey-Kramer* | *0.0003* | *0.05* | *3.25* | *7.40* | *1.26* | *9.39* |
| ***diagnosis*sector*** | ***6*** | ***0*** | ***10*** | ***2*** | *5.30* | *1.06* | *1947* | *5.02* | *<.0001* | *Tukey-Kramer* | *0.0003* | *0.05* | *3.23* | *7.38* | *1.24* | *9.37* |
| ***diagnosis*sector*** | ***7*** | ***0*** | ***8*** | ***2*** | *5.33* | *1.06* | *1947* | *5.04* | *<.0001* | *Tukey-Kramer* | *0.0003* | *0.05* | *3.26* | *7.40* | *1.27* | *9.39* |
| ***diagnosis*sector*** | ***1*** | ***1*** | ***9*** | ***2*** | *5.42* | *1.08* | *1947* | *5.02* | *<.0001* | *Tukey-Kramer* | *0.0003* | *0.05* | *3.30* | *7.54* | *1.27* | *9.57* |
| ***diagnosis*sector*** | ***4*** | ***1*** | ***10*** | ***2*** | *5.44* | *1.08* | *1947* | *5.04* | *<.0001* | *Tukey-Kramer* | *0.0003* | *0.05* | *3.32* | *7.56* | *1.29* | *9.59* |
| ***diagnosis*sector*** | ***7*** | ***1*** | ***10*** | ***2*** | *5.35* | *1.08* | *1947* | *4.96* | *<.0001* | *Tukey-Kramer* | *0.0004* | *0.05* | *3.24* | *7.47* | *1.20* | *9.50* |
| ***diagnosis*sector*** | ***1*** | ***0*** | ***8*** | ***2*** | *5.21* | *1.06* | *1947* | *4.92* | *<.0001* | *Tukey-Kramer* | *0.0005* | *0.05* | *3.13* | *7.28* | *1.14* | *9.27* |
| ***diagnosis*sector*** | ***12*** | ***0*** | ***8*** | ***2*** | *5.23* | *1.06* | *1947* | *4.95* | *<.0001* | *Tukey-Kramer* | *0.0005* | *0.05* | *3.16* | *7.30* | *1.17* | *9.29* |
| ***diagnosis*sector*** | ***8*** | ***0*** | ***9*** | ***2*** | *5.18* | *1.06* | *1947* | *4.9* | *<.0001* | *Tukey-Kramer* | *0.0006* | *0.05* | *3.11* | *7.26* | *1.12* | *9.25* |
| ***diagnosis*sector*** | ***12*** | ***2*** | ***9*** | ***2*** | *3.78* | *0.77* | *1947* | *4.89* | *<.0001* | *Tukey-Kramer* | *0.0006* | *0.05* | *2.26* | *5.29* | *0.81* | *6.74* |
| ***diagnosis*sector*** | ***2*** | ***0*** | ***9*** | ***2*** | *5.16* | *1.06* | *1947* | *4.88* | *<.0001* | *Tukey-Kramer* | *0.0007* | *0.05* | *3.08* | *7.23* | *1.09* | *9.22* |
| ***diagnosis*sector*** | ***10*** | ***1*** | ***4*** | ***1*** | *-2.72* | *0.56* | *1947* | *-4.87* | *<.0001* | *Tukey-Kramer* | *0.0007* | *0.05* | *-3.82* | *-1.63* | *-4.88* | *-0.57* |
| ***diagnosis*sector*** | ***4*** | ***1*** | ***8*** | ***2*** | *5.23* | *1.08* | *1947* | *4.85* | *<.0001* | *Tukey-Kramer* | *0.0008* | *0.05* | *3.12* | *7.35* | *1.08* | *9.38* |
| ***diagnosis*sector*** | ***6*** | ***0*** | ***8*** | ***2*** | *5.10* | *1.06* | *1947* | *4.82* | *<.0001* | *Tukey-Kramer* | *0.0009* | *0.05* | *3.02* | *7.17* | *1.03* | *9.16* |
| ***diagnosis*sector*** | ***8*** | ***0*** | ***9*** | ***0*** | *1.50* | *0.31* | *1947* | *4.8* | *<.0001* | *Tukey-Kramer* | *0.001* | *0.05* | *0.89* | *2.11* | *0.30* | *2.70* |
| ***diagnosis*sector*** | ***5*** | ***0*** | ***10*** | ***2*** | *5.05* | *1.06* | *1947* | *4.77* | *<.0001* | *Tukey-Kramer* | *0.0011* | *0.05* | *2.97* | *7.12* | *0.98* | *9.11* |
| ***diagnosis*sector*** | ***1*** | ***1*** | ***10*** | ***2*** | *5.15* | *1.08* | *1947* | *4.77* | *<.0001* | *Tukey-Kramer* | *0.0011* | *0.05* | *3.03* | *7.26* | *1.00* | *9.30* |
| ***diagnosis*sector*** | ***7*** | ***1*** | ***8*** | ***2*** | *5.14* | *1.08* | *1947* | *4.77* | *<.0001* | *Tukey-Kramer* | *0.0011* | *0.05* | *3.03* | *7.26* | *1.00* | *9.29* |
| ***diagnosis*sector*** | ***3*** | ***2*** | ***9*** | ***2*** | *3.66* | *0.77* | *1947* | *4.75* | *<.0001* | *Tukey-Kramer* | *0.0012* | *0.05* | *2.15* | *5.18* | *0.70* | *6.63* |
| ***diagnosis*sector*** | ***4*** | ***0*** | ***9*** | ***2*** | *5.01* | *1.06* | *1947* | *4.74* | *<.0001* | *Tukey-Kramer* | *0.0013* | *0.05* | *2.93* | *7.08* | *0.94* | *9.07* |
| ***diagnosis*sector*** | ***2*** | ***0*** | ***9*** | ***0*** | *1.47* | *0.31* | *1947* | *4.71* | *<.0001* | *Tukey-Kramer* | *0.0015* | *0.05* | *0.86* | *2.08* | *0.27* | *2.67* |
| ***diagnosis*sector*** | ***10*** | ***1*** | ***7*** | ***1*** | *-2.64* | *0.56* | *1947* | *-4.71* | *<.0001* | *Tukey-Kramer* | *0.0015* | *0.05* | *-3.73* | *-1.54* | *-4.79* | *-0.49* |
| ***diagnosis*sector*** | ***6*** | ***1*** | ***9*** | ***2*** | *5.07* | *1.08* | *1947* | *4.69* | *<.0001* | *Tukey-Kramer* | *0.0016* | *0.05* | *2.95* | *7.18* | *0.92* | *9.22* |
| ***diagnosis*sector*** | ***2*** | ***1*** | ***9*** | ***2*** | *5.04* | *1.08* | *1947* | *4.67* | *<.0001* | *Tukey-Kramer* | *0.0017* | *0.05* | *2.93* | *7.16* | *0.89* | *9.19* |
| ***diagnosis*sector*** | ***8*** | ***0*** | ***10*** | ***2*** | *4.91* | *1.06* | *1947* | *4.64* | *<.0001* | *Tukey-Kramer* | *0.002* | *0.05* | *2.84* | *6.98* | *0.85* | *8.97* |
| ***diagnosis*sector*** | ***12*** | ***1*** | ***9*** | ***2*** | *5.00* | *1.08* | *1947* | *4.63* | *<.0001* | *Tukey-Kramer* | *0.0021* | *0.05* | *2.88* | *7.12* | *0.85* | *9.15* |
| ***diagnosis*sector*** | ***2*** | ***0*** | ***10*** | ***2*** | *4.88* | *1.06* | *1947* | *4.62* | *<.0001* | *Tukey-Kramer* | *0.0022* | *0.05* | *2.81* | *6.96* | *0.82* | *8.95* |
| ***diagnosis*sector*** | ***10*** | ***0*** | ***11*** | ***0*** | *-1.43* | *0.31* | *1947* | *-4.58* | *<.0001* | *Tukey-Kramer* | *0.0026* | *0.05* | *-2.04* | *-0.82* | *-2.63* | *-0.23* |
| ***diagnosis*sector*** | ***5*** | ***0*** | ***8*** | ***2*** | *4.84* | *1.06* | *1947* | *4.58* | *<.0001* | *Tukey-Kramer* | *0.0027* | *0.05* | *2.77* | *6.91* | *0.78* | *8.90* |
| ***diagnosis*sector*** | ***1*** | ***1*** | ***8*** | ***2*** | *4.94* | *1.08* | *1947* | *4.57* | *<.0001* | *Tukey-Kramer* | *0.0027* | *0.05* | *2.82* | *7.06* | *0.79* | *9.09* |
| ***diagnosis*sector*** | ***5*** | ***1*** | ***9*** | ***2*** | *4.94* | *1.08* | *1947* | *4.57* | *<.0001* | *Tukey-Kramer* | *0.0027* | *0.05* | *2.82* | *7.06* | *0.79* | *9.09* |
| ***diagnosis*sector*** | ***10*** | ***2*** | ***12*** | ***2*** | *-3.50* | *0.77* | *1947* | *-4.54* | *<.0001* | *Tukey-Kramer* | *0.0032* | *0.05* | *-5.01* | *-1.99* | *-6.47* | *-0.54* |
| ***diagnosis*sector*** | ***3*** | ***1*** | ***9*** | ***2*** | *4.84* | *1.08* | *1947* | *4.49* | *<.0001* | *Tukey-Kramer* | *0.004* | *0.05* | *2.73* | *6.96* | *0.69* | *8.99* |
| ***diagnosis*sector*** | ***4*** | ***0*** | ***10*** | ***2*** | *4.74* | *1.06* | *1947* | *4.48* | *<.0001* | *Tukey-Kramer* | *0.0042* | *0.05* | *2.66* | *6.81* | *0.67* | *8.80* |
| ***diagnosis*sector*** | ***8*** | ***1*** | ***9*** | ***2*** | *4.83* | *1.08* | *1947* | *4.47* | *<.0001* | *Tukey-Kramer* | *0.0042* | *0.05* | *2.71* | *6.95* | *0.68* | *8.98* |
| ***diagnosis*sector*** | ***8*** | ***0*** | ***8*** | ***2*** | *4.70* | *1.06* | *1947* | *4.45* | *<.0001* | *Tukey-Kramer* | *0.0048* | *0.05* | *2.63* | *6.78* | *0.64* | *8.77* |
| ***diagnosis*sector*** | ***6*** | ***1*** | ***10*** | ***2*** | *4.79* | *1.08* | *1947* | *4.44* | *<.0001* | *Tukey-Kramer* | *0.0049* | *0.05* | *2.68* | *6.91* | *0.64* | *8.94* |
| ***diagnosis*sector*** | ***2*** | ***0*** | ***8*** | ***2*** | *4.67* | *1.06* | *1947* | *4.42* | *<.0001* | *Tukey-Kramer* | *0.0053* | *0.05* | *2.60* | *6.75* | *0.61* | *8.74* |
| ***diagnosis*sector*** | ***7*** | ***0*** | ***6*** | ***2*** | *4.67* | *1.06* | *1947* | *4.42* | *<.0001* | *Tukey-Kramer* | *0.0053* | *0.05* | *2.60* | *6.75* | *0.61* | *8.74* |
| ***diagnosis*sector*** | ***2*** | ***1*** | ***10*** | ***2*** | *4.77* | *1.08* | *1947* | *4.42* | *<.0001* | *Tukey-Kramer* | *0.0054* | *0.05* | *2.65* | *6.89* | *0.62* | *8.92* |
| ***diagnosis*sector*** | ***10*** | ***2*** | ***3*** | ***2*** | *-3.39* | *0.77* | *1947* | *-4.39* | *<.0001* | *Tukey-Kramer* | *0.006* | *0.05* | *-4.90* | *-1.88* | *-6.36* | *-0.43* |
| ***diagnosis*sector*** | ***11*** | ***0*** | ***9*** | ***2*** | *4.64* | *1.06* | *1947* | *4.39* | *<.0001* | *Tukey-Kramer* | *0.0061* | *0.05* | *2.57* | *6.71* | *0.58* | *8.70* |
| ***diagnosis*sector*** | ***12*** | ***1*** | ***10*** | ***2*** | *4.73* | *1.08* | *1947* | *4.38* | *<.0001* | *Tukey-Kramer* | *0.0064* | *0.05* | *2.61* | *6.85* | *0.58* | *8.88* |
| ***diagnosis*sector*** | ***1*** | ***1*** | ***10*** | ***1*** | *2.43* | *0.56* | *1947* | *4.34* | *<.0001* | *Tukey-Kramer* | *0.0074* | *0.05* | *1.33* | *3.53* | *0.28* | *4.58* |
| ***diagnosis*sector*** | ***3*** | ***0*** | ***7*** | ***0*** | *-1.36* | *0.31* | *1947* | *-4.34* | *<.0001* | *Tukey-Kramer* | *0.0075* | *0.05* | *-1.97* | *-0.74* | *-2.56* | *-0.16* |
| ***diagnosis*sector*** | ***12*** | ***0*** | ***6*** | ***2*** | *4.57* | *1.06* | *1947* | *4.33* | *<.0001* | *Tukey-Kramer* | *0.008* | *0.05* | *2.50* | *6.65* | *0.51* | *8.64* |
| ***diagnosis*sector*** | ***5*** | ***1*** | ***10*** | ***2*** | *4.66* | *1.08* | *1947* | *4.32* | *<.0001* | *Tukey-Kramer* | *0.0081* | *0.05* | *2.55* | *6.78* | *0.52* | *8.81* |
| ***diagnosis*sector*** | ***1*** | ***0*** | ***6*** | ***2*** | *4.55* | *1.06* | *1947* | *4.3* | *<.0001* | *Tukey-Kramer* | *0.0087* | *0.05* | *2.48* | *6.62* | *0.49* | *8.61* |
| ***diagnosis*sector*** | ***4*** | ***0*** | ***8*** | ***2*** | *4.53* | *1.06* | *1947* | *4.28* | *<.0001* | *Tukey-Kramer* | *0.0096* | *0.05* | *2.45* | *6.60* | *0.46* | *8.59* |
| ***diagnosis*sector*** | ***12*** | ***2*** | ***8*** | ***2*** | *3.29* | *0.77* | *1947* | *4.27* | *<.0001* | *Tukey-Kramer* | *0.01* | *0.05* | *1.78* | *4.81* | *0.33* | *6.26* |
| ***diagnosis*sector*** | ***6*** | ***1*** | ***8*** | ***2*** | *4.59* | *1.08* | *1947* | *4.25* | *<.0001* | *Tukey-Kramer* | *0.0109* | *0.05* | *2.47* | *6.70* | *0.44* | *8.74* |
| ***diagnosis*sector*** | ***4*** | ***1*** | ***6*** | ***2*** | *4.58* | *1.08* | *1947* | *4.24* | *<.0001* | *Tukey-Kramer* | *0.0113* | *0.05* | *2.46* | *6.69* | *0.43* | *8.73* |
| ***diagnosis*sector*** | ***4*** | ***0*** | ***9*** | ***0*** | *1.32* | *0.31* | *1947* | *4.24* | *<.0001* | *Tukey-Kramer* | *0.0115* | *0.05* | *0.71* | *1.94* | *0.12* | *2.52* |
| ***diagnosis*sector*** | ***3*** | ***1*** | ***10*** | ***2*** | *4.57* | *1.08* | *1947* | *4.23* | *<.0001* | *Tukey-Kramer* | *0.0116* | *0.05* | *2.45* | *6.69* | *0.42* | *8.72* |
| ***diagnosis*sector*** | ***2*** | ***1*** | ***8*** | ***2*** | *4.56* | *1.08* | *1947* | *4.23* | *<.0001* | *Tukey-Kramer* | *0.012* | *0.05* | *2.45* | *6.68* | *0.41* | *8.71* |
| ***diagnosis*sector*** | ***8*** | ***1*** | ***10*** | ***2*** | *4.56* | *1.08* | *1947* | *4.22* | *<.0001* | *Tukey-Kramer* | *0.0122* | *0.05* | *2.44* | *6.67* | *0.41* | *8.71* |
| ***diagnosis*sector*** | ***3*** | ***0*** | ***9*** | ***2*** | *4.46* | *1.06* | *1947* | *4.21* | *<.0001* | *Tukey-Kramer* | *0.0125* | *0.05* | *2.38* | *6.53* | *0.39* | *8.52* |
| ***diagnosis*sector*** | ***4*** | ***2*** | ***9*** | ***2*** | *3.25* | *0.77* | *1947* | *4.21* | *<.0001* | *Tukey-Kramer* | *0.0129* | *0.05* | *1.73* | *4.76* | *0.28* | *6.21* |
| ***diagnosis*sector*** | ***6*** | ***0*** | ***6*** | ***2*** | *4.44* | *1.06* | *1947* | *4.2* | *<.0001* | *Tukey-Kramer* | *0.0133* | *0.05* | *2.37* | *6.51* | *0.38* | *8.50* |
| ***diagnosis*sector*** | ***12*** | ***1*** | ***8*** | ***2*** | *4.52* | *1.08* | *1947* | *4.19* | *<.0001* | *Tukey-Kramer* | *0.014* | *0.05* | *2.40* | *6.64* | *0.37* | *8.67* |
| ***diagnosis*sector*** | ***1*** | ***2*** | ***9*** | ***2*** | *3.22* | *0.77* | *1947* | *4.18* | *<.0001* | *Tukey-Kramer* | *0.0146* | *0.05* | *1.71* | *4.73* | *0.26* | *6.19* |
| ***diagnosis*sector*** | ***7*** | ***1*** | ***6*** | ***2*** | *4.49* | *1.08* | *1947* | *4.16* | *<.0001* | *Tukey-Kramer* | *0.0157* | *0.05* | *2.37* | *6.61* | *0.34* | *8.64* |
| ***diagnosis*sector*** | ***4*** | ***1*** | ***9*** | ***1*** | *2.32* | *0.56* | *1947* | *4.15* | *<.0001* | *Tukey-Kramer* | *0.0162* | *0.05* | *1.23* | *3.42* | *0.17* | *4.47* |
| ***diagnosis*sector*** | ***11*** | ***0*** | ***10*** | ***2*** | *4.37* | *1.06* | *1947* | *4.13* | *<.0001* | *Tukey-Kramer* | *0.0175* | *0.05* | *2.29* | *6.44* | *0.30* | *8.43* |
| ***diagnosis*sector*** | ***5*** | ***1*** | ***8*** | ***2*** | *4.46* | *1.08* | *1947* | *4.13* | *<.0001* | *Tukey-Kramer* | *0.0176* | *0.05* | *2.34* | *6.57* | *0.31* | *8.61* |
| ***diagnosis*sector*** | ***3*** | ***2*** | ***8*** | ***2*** | *3.18* | *0.77* | *1947* | *4.13* | *<.0001* | *Tukey-Kramer* | *0.0178* | *0.05* | *1.67* | *4.70* | *0.22* | *6.15* |
| ***diagnosis*sector*** | ***3*** | ***1*** | ***8*** | ***2*** | *4.36* | *1.08* | *1947* | *4.04* | *<.0001* | *Tukey-Kramer* | *0.0246* | *0.05* | *2.24* | *6.48* | *0.21* | *8.51* |
| ***diagnosis*sector*** | ***8*** | ***1*** | ***8*** | ***2*** | *4.35* | *1.08* | *1947* | *4.03* | *<.0001* | *Tukey-Kramer* | *0.0258* | *0.05* | *2.23* | *6.47* | *0.20* | *8.50* |
| ***diagnosis*sector*** | ***2*** | ***2*** | ***9*** | ***2*** | *3.10* | *0.77* | *1947* | *4.02* | *<.0001* | *Tukey-Kramer* | *0.0264* | *0.05* | *1.59* | *4.62* | *0.14* | *6.07* |
| ***diagnosis*sector*** | ***12*** | ***0*** | ***3*** | ***0*** | *1.25* | *0.31* | *1947* | *4.02* | *<.0001* | *Tukey-Kramer* | *0.027* | *0.05* | *0.64* | *1.87* | *0.05* | *2.45* |
| ***diagnosis*sector*** | ***10*** | ***0*** | ***3*** | ***0*** | *-1.25* | *0.31* | *1947* | *-3.99* | *<.0001* | *Tukey-Kramer* | *0.0294* | *0.05* | *-1.86* | *-0.63* | *-2.45* | *-0.05* |
| ***diagnosis*sector*** | ***7*** | ***1*** | ***9*** | ***1*** | *2.23* | *0.56* | *1947* | *3.99* | *<.0001* | *Tukey-Kramer* | *0.0295* | *0.05* | *1.14* | *3.33* | *0.08* | *4.39* |
| ***diagnosis*sector*** | ***1*** | ***1*** | ***6*** | ***2*** | *4.28* | *1.08* | *1947* | *3.97* | *<.0001* | *Tukey-Kramer* | *0.0323* | *0.05* | *2.17* | *6.40* | *0.13* | *8.43* |
| ***diagnosis*sector*** | ***5*** | ***0*** | ***6*** | ***2*** | *4.18* | *1.06* | *1947* | *3.96* | *<.0001* | *Tukey-Kramer* | *0.0334* | *0.05* | *2.11* | *6.26* | *0.12* | *8.25* |
| ***diagnosis*sector*** | ***3*** | ***0*** | ***10*** | ***2*** | *4.18* | *1.06* | *1947* | *3.96* | *<.0001* | *Tukey-Kramer* | *0.0336* | *0.05* | *2.11* | *6.26* | *0.12* | *8.25* |
| ***diagnosis*sector*** | ***1*** | ***0*** | ***3*** | ***0*** | *1.23* | *0.31* | *1947* | *3.94* | *<.0001* | *Tukey-Kramer* | *0.0352* | *0.05* | *0.62* | *1.84* | *0.03* | *2.43* |
| ***diagnosis*sector*** | ***11*** | ***0*** | ***8*** | ***2*** | *4.16* | *1.06* | *1947* | *3.93* | *<.0001* | *Tukey-Kramer* | *0.0365* | *0.05* | *2.08* | *6.23* | *0.09* | *8.22* |
| ***diagnosis*sector*** | ***7*** | ***0*** | ***7*** | ***2*** | *4.08* | *1.06* | *1947* | *3.86* | *0.0001* | *Tukey-Kramer* | *0.0478* | *0.05* | *2.00* | *6.15* | *0.01* | *8.14* |
| ***diagnosis*sector*** | ***10*** | ***2*** | ***4*** | ***2*** | *-2.97* | *0.77* | *1947* | *-3.85* | *0.0001* | *Tukey-Kramer* | *0.0483* | *0.05* | *-4.49* | *-1.46* | *-5.94* | *-0.01* |
| ***diagnosis*sector*** | ***1*** | ***0*** | ***10*** | ***0*** | *2.48* | *0.31* | *1947* | *7.94* | *<.0001* | *Tukey-Kramer* | *<.0001* | *0.05* | *1.87* | *3.09* | *1.28* | *3.68* |
| ***diagnosis*sector*** | ***1*** | ***0*** | ***9*** | ***0*** | *2.00* | *0.31* | *1947* | *6.41* | *<.0001* | *Tukey-Kramer* | *<.0001* | *0.05* | *1.39* | *2.61* | *0.80* | *3.20* |
| ***diagnosis*sector*** | ***1*** | ***0*** | ***9*** | ***2*** | *5.69* | *1.06* | *1947* | *5.38* | *<.0001* | *Tukey-Kramer* | *<.0001* | *0.05* | *3.61* | *7.76* | *1.62* | *9.75* |
| ***diagnosis*sector*** | ***10*** | ***0*** | ***12*** | ***0*** | *-2.50* | *0.31* | *1947* | *-8.01* | *<.0001* | *Tukey-Kramer* | *<.0001* | *0.05* | *-3.11* | *-1.89* | *-3.70* | *-1.30* |
| ***diagnosis*sector*** | ***10*** | ***0*** | ***2*** | ***0*** | *-1.95* | *0.31* | *1947* | *-6.23* | *<.0001* | *Tukey-Kramer* | *<.0001* | *0.05* | *-2.56* | *-1.33* | *-3.15* | *-0.75* |
| ***diagnosis*sector*** | ***10*** | ***0*** | ***4*** | ***0*** | *-1.80* | *0.31* | *1947* | *-5.76* | *<.0001* | *Tukey-Kramer* | *<.0001* | *0.05* | *-2.41* | *-1.19* | *-3.00* | *-0.60* |
| ***diagnosis*sector*** | ***10*** | ***0*** | ***5*** | ***0*** | *-2.11* | *0.31* | *1947* | *-6.76* | *<.0001* | *Tukey-Kramer* | *<.0001* | *0.05* | *-2.73* | *-1.50* | *-3.31* | *-0.91* |
| ***diagnosis*sector*** | ***10*** | ***0*** | ***6*** | ***0*** | *-2.37* | *0.31* | *1947* | *-7.58* | *<.0001* | *Tukey-Kramer* | *<.0001* | *0.05* | *-2.98* | *-1.76* | *-3.57* | *-1.17* |
| ***diagnosis*sector*** | ***10*** | ***0*** | ***7*** | ***0*** | *-2.60* | *0.31* | *1947* | *-8.33* | *<.0001* | *Tukey-Kramer* | *<.0001* | *0.05* | *-3.22* | *-1.99* | *-3.80* | *-1.40* |
| ***diagnosis*sector*** | ***10*** | ***0*** | ***8*** | ***0*** | *-1.97* | *0.31* | *1947* | *-6.32* | *<.0001* | *Tukey-Kramer* | *<.0001* | *0.05* | *-2.59* | *-1.36* | *-3.18* | *-0.77* |
| ***diagnosis*sector*** | ***12*** | ***0*** | ***9*** | ***0*** | *2.02* | *0.31* | *1947* | *6.48* | *<.0001* | *Tukey-Kramer* | *<.0001* | *0.05* | *1.41* | *2.64* | *0.82* | *3.23* |
| ***diagnosis*sector*** | ***12*** | ***0*** | ***9*** | ***2*** | *5.71* | *1.06* | *1947* | *5.4* | *<.0001* | *Tukey-Kramer* | *<.0001* | *0.05* | *3.64* | *7.78* | *1.65* | *9.77* |
| ***diagnosis*sector*** | ***6*** | ***0*** | ***9*** | ***0*** | *1.89* | *0.31* | *1947* | *6.06* | *<.0001* | *Tukey-Kramer* | *<.0001* | *0.05* | *1.28* | *2.50* | *0.69* | *3.09* |
| ***diagnosis*sector*** | ***6*** | ***0*** | ***9*** | ***2*** | *5.58* | *1.06* | *1947* | *5.28* | *<.0001* | *Tukey-Kramer* | *<.0001* | *0.05* | *3.50* | *7.65* | *1.51* | *9.64* |
| ***diagnosis*sector*** | ***7*** | ***0*** | ***9*** | ***0*** | *2.13* | *0.31* | *1947* | *6.81* | *<.0001* | *Tukey-Kramer* | *<.0001* | *0.05* | *1.51* | *2.74* | *0.93* | *3.33* |
| ***diagnosis*sector*** | ***7*** | ***0*** | ***9*** | ***2*** | *5.81* | *1.06* | *1947* | *5.5* | *<.0001* | *Tukey-Kramer* | *<.0001* | *0.05* | *3.74* | *7.89* | *1.75* | *9.88* |
| ***diagnosis*sector*** | ***4*** | ***1*** | ***9*** | ***2*** | *5.71* | *1.08* | *1947* | *5.29* | *<.0001* | *Tukey-Kramer* | *<.0001* | *0.05* | *3.60* | *7.83* | *1.56* | *9.86* |
| ***diagnosis*** |  | ***0*** |  | ***2*** | *2.90* | *0.89* | *1947* | *3.24* | *0.0012* | *Tukey-Kramer* | *0.0035* | *0.05* | *1.14* | *4.65* | *0.80* | *5.00* |
| ***diagnosis*** |  | ***1*** |  | ***2*** | *2.67* | *0.87* | *1947* | *3.09* | *0.002* | *Tukey-Kramer* | *0.0058* | *0.05* | *0.98* | *4.37* | *0.64* | *4.70* |

b)

| **ICP** | | | | | | | | | | | | | | | | |
| --- | --- | --- | --- | --- | --- | --- | --- | --- | --- | --- | --- | --- | --- | --- | --- | --- |
| **Differences of Least Squares Means** | | | | | | | | | | | | | | | | |
| **Effect** | **Sector** | **Glk** | **Sector** | **Glk** | **Estimate** | **Standard** | **DF** | **t Value** | **Pr > \|t\|** | **Adjustment** | **Adj P** | **Alpha** | **Lower** | **Upper** | **Adj Lower** | **Adj Upper** |
|  |  |  |  |  |  | **Error** |  |  |  |  |  |  |  |  |  |  |
| **sector** | **10** |  | **5** |  | 1.30 | 0.30 | 1947 | 4.30 | <.0001 | Tukey-Kramer | 0.0011 | 0.05 | 0.71 | 1.89 | 0.31 | 2.29 |
| **sector** | **2** |  | **6** |  | 1.24 | 0.30 | 1947 | 4.09 | <.0001 | Tukey-Kramer | 0.0026 | 0.05 | 0.64 | 1.83 | 0.25 | 2.22 |
| **sector** | **10** |  | **4** |  | -1.20 | 0.30 | 1947 | -3.97 | <.0001 | Tukey-Kramer | 0.0042 | 0.05 | -1.79 | -0.61 | -2.19 | -0.21 |
| **sector** | **10** |  | **2** |  | 1.17 | 0.30 | 1947 | 3.88 | 0.0001 | Tukey-Kramer | 0.0062 | 0.05 | 0.58 | 1.76 | 0.18 | 2.16 |
| **sector** | **5** |  | **6** |  | 1.11 | 0.30 | 1947 | 3.67 | 0.0002 | Tukey-Kramer | 0.0133 | 0.05 | 0.52 | 1.70 | 0.12 | 2.09 |
| **sector** | **1** |  | **10** |  | -1.82 | 0.30 | 1947 | -6.03 | <.0001 | Tukey-Kramer | <.0001 | 0.05 | -2.41 | -1.23 | -2.81 | -0.83 |
| **sector** | **1** |  | **3** |  | -2.61 | 0.30 | 1947 | -8.63 | <.0001 | Tukey-Kramer | <.0001 | 0.05 | -3.20 | -2.01 | -3.59 | -1.62 |
| **sector** | **1** |  | **4** |  | -3.02 | 0.30 | 1947 | -10.00 | <.0001 | Tukey-Kramer | <.0001 | 0.05 | -3.61 | -2.43 | -4.01 | -2.03 |
| **sector** | **1** |  | **9** |  | -2.19 | 0.30 | 1947 | -7.25 | <.0001 | Tukey-Kramer | <.0001 | 0.05 | -2.78 | -1.60 | -3.18 | -1.20 |
| **sector** | **10** |  | **11** |  | 1.65 | 0.30 | 1947 | 5.48 | <.0001 | Tukey-Kramer | <.0001 | 0.05 | 1.06 | 2.24 | 0.67 | 2.64 |
| **sector** | **10** |  | **12** |  | 1.72 | 0.30 | 1947 | 5.71 | <.0001 | Tukey-Kramer | <.0001 | 0.05 | 1.13 | 2.32 | 0.74 | 2.71 |
| **sector** | **10** |  | **6** |  | 2.41 | 0.30 | 1947 | 7.97 | <.0001 | Tukey-Kramer | <.0001 | 0.05 | 1.81 | 3.00 | 1.42 | 3.39 |
| **sector** | **10** |  | **7** |  | 2.13 | 0.30 | 1947 | 7.05 | <.0001 | Tukey-Kramer | <.0001 | 0.05 | 1.53 | 2.72 | 1.14 | 3.11 |
| **sector** | **10** |  | **8** |  | 1.77 | 0.30 | 1947 | 5.85 | <.0001 | Tukey-Kramer | <.0001 | 0.05 | 1.18 | 2.36 | 0.78 | 2.75 |
| **sector** | **11** |  | **3** |  | -2.44 | 0.30 | 1947 | -8.08 | <.0001 | Tukey-Kramer | <.0001 | 0.05 | -3.03 | -1.85 | -3.43 | -1.45 |
| **sector** | **11** |  | **4** |  | -2.85 | 0.30 | 1947 | -9.45 | <.0001 | Tukey-Kramer | <.0001 | 0.05 | -3.44 | -2.26 | -3.84 | -1.86 |
| **sector** | **11** |  | **9** |  | -2.02 | 0.30 | 1947 | -6.70 | <.0001 | Tukey-Kramer | <.0001 | 0.05 | -2.61 | -1.43 | -3.01 | -1.03 |
| **sector** | **12** |  | **3** |  | -2.51 | 0.30 | 1947 | -8.31 | <.0001 | Tukey-Kramer | <.0001 | 0.05 | -3.10 | -1.92 | -3.50 | -1.52 |
| **sector** | **12** |  | **4** |  | -2.92 | 0.30 | 1947 | -9.68 | <.0001 | Tukey-Kramer | <.0001 | 0.05 | -3.51 | -2.33 | -3.91 | -1.94 |
| **sector** | **12** |  | **9** |  | -2.09 | 0.30 | 1947 | -6.93 | <.0001 | Tukey-Kramer | <.0001 | 0.05 | -2.68 | -1.50 | -3.08 | -1.11 |
| **sector** | **2** |  | **3** |  | -1.95 | 0.30 | 1947 | -6.48 | <.0001 | Tukey-Kramer | <.0001 | 0.05 | -2.55 | -1.36 | -2.94 | -0.97 |
| **sector** | **2** |  | **4** |  | -2.37 | 0.30 | 1947 | -7.85 | <.0001 | Tukey-Kramer | <.0001 | 0.05 | -2.96 | -1.78 | -3.36 | -1.38 |
| **sector** | **2** |  | **9** |  | -1.54 | 0.30 | 1947 | -5.10 | <.0001 | Tukey-Kramer | <.0001 | 0.05 | -2.13 | -0.95 | -2.53 | -0.55 |
| **sector** | **3** |  | **5** |  | 2.08 | 0.30 | 1947 | 6.90 | <.0001 | Tukey-Kramer | <.0001 | 0.05 | 1.49 | 2.67 | 1.10 | 3.07 |
| **sector** | **3** |  | **6** |  | 3.19 | 0.30 | 1947 | 10.57 | <.0001 | Tukey-Kramer | <.0001 | 0.05 | 2.60 | 3.78 | 2.20 | 4.18 |
| **sector** | **3** |  | **7** |  | 2.91 | 0.30 | 1947 | 9.65 | <.0001 | Tukey-Kramer | <.0001 | 0.05 | 2.32 | 3.50 | 1.92 | 3.90 |
| **sector** | **3** |  | **8** |  | 2.55 | 0.30 | 1947 | 8.46 | <.0001 | Tukey-Kramer | <.0001 | 0.05 | 1.96 | 3.14 | 1.56 | 3.54 |
| **sector** | **4** |  | **5** |  | 2.50 | 0.30 | 1947 | 8.27 | <.0001 | Tukey-Kramer | <.0001 | 0.05 | 1.90 | 3.09 | 1.51 | 3.48 |
| **sector** | **4** |  | **6** |  | 3.60 | 0.30 | 1947 | 11.94 | <.0001 | Tukey-Kramer | <.0001 | 0.05 | 3.01 | 4.20 | 2.62 | 4.59 |
| **sector** | **4** |  | **7** |  | 3.33 | 0.30 | 1947 | 11.02 | <.0001 | Tukey-Kramer | <.0001 | 0.05 | 2.73 | 3.92 | 2.34 | 4.31 |
| **sector** | **4** |  | **8** |  | 2.97 | 0.30 | 1947 | 9.83 | <.0001 | Tukey-Kramer | <.0001 | 0.05 | 2.37 | 3.56 | 1.98 | 3.95 |
| **sector** | **5** |  | **9** |  | -1.67 | 0.30 | 1947 | -5.52 | <.0001 | Tukey-Kramer | <.0001 | 0.05 | -2.26 | -1.07 | -2.65 | -0.68 |
| **sector** | **6** |  | **9** |  | -2.77 | 0.30 | 1947 | -9.19 | <.0001 | Tukey-Kramer | <.0001 | 0.05 | -3.37 | -2.18 | -3.76 | -1.79 |
| **sector** | **7** |  | **9** |  | -2.50 | 0.30 | 1947 | -8.27 | <.0001 | Tukey-Kramer | <.0001 | 0.05 | -3.09 | -1.90 | -3.48 | -1.51 |
| **sector** | **8** |  | **9** |  | -2.14 | 0.30 | 1947 | -7.08 | <.0001 | Tukey-Kramer | <.0001 | 0.05 | -2.73 | -1.54 | -3.12 | -1.15 |
| **diagnosis*sector** | **12** | **2** | **3** | **2** | -3.63 | 0.70 | 1947 | -5.21 | <.0001 | Tukey-Kramer | 0.0001 | 0.05 | -4.99 | -2.26 | -6.31 | -0.95 |
| **diagnosis*sector** | **1** | **1** | **4** | **1** | -2.60 | 0.51 | 1947 | -5.14 | <.0001 | Tukey-Kramer | 0.0002 | 0.05 | -3.59 | -1.61 | -4.54 | -0.66 |
| **diagnosis*sector** | **4** | **1** | **7** | **1** | 2.57 | 0.51 | 1947 | 5.09 | <.0001 | Tukey-Kramer | 0.0002 | 0.05 | 1.58 | 3.56 | 0.63 | 4.51 |
| **diagnosis*sector** | **1** | **0** | **10** | **0** | -1.42 | 0.28 | 1947 | -5.03 | <.0001 | Tukey-Kramer | 0.0003 | 0.05 | -1.97 | -0.86 | -2.50 | -0.33 |
| **diagnosis*sector** | **9** | **0** | **6** | **2** | 4.53 | 0.90 | 1947 | 5.04 | <.0001 | Tukey-Kramer | 0.0003 | 0.05 | 2.77 | 6.29 | 1.08 | 7.98 |
| **diagnosis*sector** | **11** | **1** | **4** | **1** | -2.55 | 0.51 | 1947 | -5.04 | <.0001 | Tukey-Kramer | 0.0003 | 0.05 | -3.54 | -1.56 | -4.49 | -0.61 |
| **diagnosis*sector** | **4** | **2** | **5** | **2** | 3.52 | 0.70 | 1947 | 5.05 | <.0001 | Tukey-Kramer | 0.0003 | 0.05 | 2.15 | 4.88 | 0.84 | 6.19 |
| **diagnosis*sector** | **4** | **1** | **6** | **2** | 4.61 | 0.92 | 1947 | 5.00 | <.0001 | Tukey-Kramer | 0.0004 | 0.05 | 2.80 | 6.42 | 1.07 | 8.16 |
| **diagnosis*sector** | **4** | **0** | **8** | **2** | 4.44 | 0.90 | 1947 | 4.94 | <.0001 | Tukey-Kramer | 0.0005 | 0.05 | 2.68 | 6.20 | 0.99 | 7.89 |
| **diagnosis*sector** | **10** | **2** | **6** | **2** | 3.43 | 0.70 | 1947 | 4.93 | <.0001 | Tukey-Kramer | 0.0005 | 0.05 | 2.07 | 4.80 | 0.76 | 6.11 |
| **diagnosis*sector** | **3** | **1** | **6** | **1** | 2.47 | 0.51 | 1947 | 4.89 | <.0001 | Tukey-Kramer | 0.0006 | 0.05 | 1.48 | 3.46 | 0.53 | 4.41 |
| **diagnosis*sector** | **11** | **2** | **3** | **2** | -3.41 | 0.70 | 1947 | -4.89 | <.0001 | Tukey-Kramer | 0.0006 | 0.05 | -4.77 | -2.04 | -6.08 | -0.73 |
| **diagnosis*sector** | **3** | **0** | **7** | **2** | 4.34 | 0.90 | 1947 | 4.83 | <.0001 | Tukey-Kramer | 0.0008 | 0.05 | 2.58 | 6.10 | 0.89 | 7.79 |
| **diagnosis*sector** | **10** | **0** | **11** | **0** | 1.36 | 0.28 | 1947 | 4.82 | <.0001 | Tukey-Kramer | 0.0009 | 0.05 | 0.81 | 1.91 | 0.28 | 2.44 |
| **diagnosis*sector** | **9** | **1** | **6** | **2** | 4.44 | 0.92 | 1947 | 4.81 | <.0001 | Tukey-Kramer | 0.0009 | 0.05 | 2.63 | 6.25 | 0.89 | 7.98 |
| **diagnosis*sector** | **1** | **1** | **9** | **1** | -2.42 | 0.51 | 1947 | -4.80 | <.0001 | Tukey-Kramer | 0.001 | 0.05 | -3.42 | -1.43 | -4.37 | -0.48 |
| **diagnosis*sector** | **10** | **0** | **6** | **2** | 4.28 | 0.90 | 1947 | 4.77 | <.0001 | Tukey-Kramer | 0.0011 | 0.05 | 2.52 | 6.05 | 0.83 | 7.73 |
| **diagnosis*sector** | **7** | **1** | **9** | **1** | -2.40 | 0.51 | 1947 | -4.74 | <.0001 | Tukey-Kramer | 0.0012 | 0.05 | -3.39 | -1.41 | -4.34 | -0.46 |
| **diagnosis*sector** | **7** | **2** | **9** | **2** | -3.30 | 0.70 | 1947 | -4.75 | <.0001 | Tukey-Kramer | 0.0012 | 0.05 | -4.67 | -1.94 | -5.98 | -0.63 |
| **diagnosis*sector** | **12** | **1** | **4** | **1** | -2.39 | 0.51 | 1947 | -4.73 | <.0001 | Tukey-Kramer | 0.0014 | 0.05 | -3.38 | -1.40 | -4.33 | -0.45 |
| **diagnosis*sector** | **11** | **1** | **9** | **1** | -2.37 | 0.51 | 1947 | -4.70 | <.0001 | Tukey-Kramer | 0.0015 | 0.05 | -3.36 | -1.38 | -4.32 | -0.43 |
| **diagnosis*sector** | **4** | **0** | **6** | **1** | 3.47 | 0.74 | 1947 | 4.69 | <.0001 | Tukey-Kramer | 0.0016 | 0.05 | 2.02 | 4.92 | 0.63 | 6.31 |
| **diagnosis*sector** | **5** | **0** | **9** | **0** | -1.31 | 0.28 | 1947 | -4.65 | <.0001 | Tukey-Kramer | 0.0019 | 0.05 | -1.87 | -0.76 | -2.40 | -0.23 |
| **diagnosis*sector** | **10** | **0** | **2** | **0** | 1.30 | 0.28 | 1947 | 4.61 | <.0001 | Tukey-Kramer | 0.0023 | 0.05 | 0.75 | 1.85 | 0.22 | 2.38 |
| **diagnosis*sector** | **10** | **0** | **8** | **0** | 1.29 | 0.28 | 1947 | 4.58 | <.0001 | Tukey-Kramer | 0.0026 | 0.05 | 0.74 | 1.85 | 0.21 | 2.38 |
| **diagnosis*sector** | **3** | **0** | **8** | **2** | 4.10 | 0.90 | 1947 | 4.56 | <.0001 | Tukey-Kramer | 0.0028 | 0.05 | 2.34 | 5.86 | 0.65 | 7.55 |
| **diagnosis*sector** | **9** | **0** | **7** | **2** | 4.09 | 0.90 | 1947 | 4.56 | <.0001 | Tukey-Kramer | 0.0029 | 0.05 | 2.33 | 5.85 | 0.64 | 7.54 |
| **diagnosis*sector** | **4** | **1** | **7** | **2** | 4.17 | 0.92 | 1947 | 4.52 | <.0001 | Tukey-Kramer | 0.0034 | 0.05 | 2.36 | 5.98 | 0.63 | 7.72 |
| **diagnosis*sector** | **3** | **1** | **6** | **2** | 4.12 | 0.92 | 1947 | 4.47 | <.0001 | Tukey-Kramer | 0.0044 | 0.05 | 2.31 | 5.93 | 0.57 | 7.67 |
| **diagnosis*sector** | **3** | **2** | **5** | **2** | 3.11 | 0.70 | 1947 | 4.46 | <.0001 | Tukey-Kramer | 0.0045 | 0.05 | 1.74 | 4.47 | 0.43 | 5.78 |
| **diagnosis*sector** | **10** | **1** | **6** | **1** | 2.24 | 0.51 | 1947 | 4.42 | <.0001 | Tukey-Kramer | 0.0053 | 0.05 | 1.24 | 3.23 | 0.29 | 4.18 |
| **diagnosis*sector** | **8** | **2** | **9** | **2** | -3.06 | 0.70 | 1947 | -4.40 | <.0001 | Tukey-Kramer | 0.0058 | 0.05 | -4.43 | -1.70 | -5.74 | -0.39 |
| **diagnosis*sector** | **12** | **1** | **9** | **1** | -2.21 | 0.51 | 1947 | -4.38 | <.0001 | Tukey-Kramer | 0.0063 | 0.05 | -3.20 | -1.22 | -4.16 | -0.27 |
| **diagnosis*sector** | **9** | **1** | **7** | **2** | 4.00 | 0.92 | 1947 | 4.34 | <.0001 | Tukey-Kramer | 0.0076 | 0.05 | 2.19 | 5.81 | 0.45 | 7.55 |
| **diagnosis*sector** | **10** | **2** | **7** | **2** | 3.00 | 0.70 | 1947 | 4.30 | <.0001 | Tukey-Kramer | 0.0087 | 0.05 | 1.63 | 4.36 | 0.32 | 5.67 |
| **diagnosis*sector** | **4** | **0** | **1** | **2** | 3.85 | 0.90 | 1947 | 4.29 | <.0001 | Tukey-Kramer | 0.0091 | 0.05 | 2.09 | 5.61 | 0.40 | 7.30 |
| **diagnosis*sector** | **9** | **0** | **8** | **2** | 3.85 | 0.90 | 1947 | 4.29 | <.0001 | Tukey-Kramer | 0.0093 | 0.05 | 2.09 | 5.61 | 0.40 | 7.30 |
| **diagnosis*sector** | **10** | **0** | **7** | **2** | 3.85 | 0.90 | 1947 | 4.29 | <.0001 | Tukey-Kramer | 0.0094 | 0.05 | 2.09 | 5.61 | 0.40 | 7.30 |
| **diagnosis*sector** | **4** | **1** | **8** | **2** | 3.93 | 0.92 | 1947 | 4.26 | <.0001 | Tukey-Kramer | 0.0103 | 0.05 | 2.12 | 5.74 | 0.39 | 7.48 |
| **diagnosis*sector** | **3** | **0** | **6** | **1** | 3.13 | 0.74 | 1947 | 4.23 | <.0001 | Tukey-Kramer | 0.0117 | 0.05 | 1.68 | 4.58 | 0.29 | 5.97 |
| **diagnosis*sector** | **10** | **1** | **6** | **2** | 3.88 | 0.92 | 1947 | 4.21 | <.0001 | Tukey-Kramer | 0.0128 | 0.05 | 2.07 | 5.69 | 0.34 | 7.43 |
| **diagnosis*sector** | **4** | **0** | **1** | **1** | 3.10 | 0.74 | 1947 | 4.19 | <.0001 | Tukey-Kramer | 0.0136 | 0.05 | 1.65 | 4.55 | 0.26 | 5.94 |
| **diagnosis*sector** | **1** | **1** | **3** | **1** | -2.11 | 0.51 | 1947 | -4.17 | <.0001 | Tukey-Kramer | 0.015 | 0.05 | -3.10 | -1.12 | -4.05 | -0.16 |
| **diagnosis*sector** | **4** | **0** | **7** | **1** | 3.07 | 0.74 | 1947 | 4.16 | <.0001 | Tukey-Kramer | 0.0158 | 0.05 | 1.62 | 4.52 | 0.23 | 5.91 |
| **diagnosis*sector** | **6** | **1** | **4** | **2** | -3.82 | 0.92 | 1947 | -4.15 | <.0001 | Tukey-Kramer | 0.0164 | 0.05 | -5.63 | -2.02 | -7.37 | -0.28 |
| **diagnosis*sector** | **2** | **1** | **4** | **1** | -2.09 | 0.51 | 1947 | -4.14 | <.0001 | Tukey-Kramer | 0.0166 | 0.05 | -3.08 | -1.10 | -4.04 | -0.15 |
| **diagnosis*sector** | **2** | **2** | **4** | **2** | -2.88 | 0.70 | 1947 | -4.14 | <.0001 | Tukey-Kramer | 0.0169 | 0.05 | -4.25 | -1.52 | -5.56 | -0.21 |
| **diagnosis*sector** | **4** | **0** | **11** | **1** | 3.05 | 0.74 | 1947 | 4.13 | <.0001 | Tukey-Kramer | 0.0178 | 0.05 | 1.60 | 4.50 | 0.21 | 5.89 |
| **diagnosis*sector** | **3** | **1** | **7** | **1** | 2.08 | 0.51 | 1947 | 4.11 | <.0001 | Tukey-Kramer | 0.0186 | 0.05 | 1.09 | 3.07 | 0.14 | 4.02 |
| **diagnosis*sector** | **4** | **1** | **5** | **1** | 2.07 | 0.51 | 1947 | 4.11 | <.0001 | Tukey-Kramer | 0.0192 | 0.05 | 1.08 | 3.07 | 0.13 | 4.02 |
| **diagnosis*sector** | **4** | **0** | **12** | **2** | 3.68 | 0.90 | 1947 | 4.10 | <.0001 | Tukey-Kramer | 0.0196 | 0.05 | 1.92 | 5.44 | 0.23 | 7.13 |
| **diagnosis*sector** | **9** | **1** | **8** | **2** | 3.76 | 0.92 | 1947 | 4.08 | <.0001 | Tukey-Kramer | 0.0216 | 0.05 | 1.95 | 5.57 | 0.21 | 7.31 |
| **diagnosis*sector** | **11** | **1** | **3** | **1** | -2.06 | 0.51 | 1947 | -4.07 | <.0001 | Tukey-Kramer | 0.0221 | 0.05 | -3.05 | -1.07 | -4.00 | -0.11 |
| **diagnosis*sector** | **10** | **0** | **8** | **2** | 3.61 | 0.90 | 1947 | 4.02 | <.0001 | Tukey-Kramer | 0.0268 | 0.05 | 1.85 | 5.37 | 0.16 | 7.06 |
| **diagnosis*sector** | **3** | **1** | **7** | **2** | 3.68 | 0.92 | 1947 | 3.99 | <.0001 | Tukey-Kramer | 0.0295 | 0.05 | 1.87 | 5.49 | 0.14 | 7.23 |
| **diagnosis*sector** | **10** | **2** | **8** | **2** | 2.76 | 0.70 | 1947 | 3.96 | <.0001 | Tukey-Kramer | 0.0333 | 0.05 | 1.39 | 4.12 | 0.08 | 5.43 |
| **diagnosis*sector** | **4** | **1** | **8** | **1** | 1.98 | 0.51 | 1947 | 3.92 | <.0001 | Tukey-Kramer | 0.0382 | 0.05 | 0.99 | 2.97 | 0.04 | 3.92 |
| **diagnosis*sector** | **3** | **0** | **1** | **2** | 3.51 | 0.90 | 1947 | 3.92 | <.0001 | Tukey-Kramer | 0.0389 | 0.05 | 1.75 | 5.28 | 0.06 | 6.96 |
| **diagnosis*sector** | **4** | **0** | **12** | **1** | 2.89 | 0.74 | 1947 | 3.91 | <.0001 | Tukey-Kramer | 0.0398 | 0.05 | 1.44 | 4.34 | 0.05 | 5.73 |
| **diagnosis*sector** | **9** | **0** | **6** | **1** | 2.88 | 0.74 | 1947 | 3.90 | 0.0001 | Tukey-Kramer | 0.0415 | 0.05 | 1.43 | 4.33 | 0.04 | 5.72 |
| **diagnosis*sector** | **4** | **0** | **11** | **2** | 3.46 | 0.90 | 1947 | 3.85 | 0.0001 | Tukey-Kramer | 0.0485 | 0.05 | 1.70 | 5.22 | 0.01 | 6.91 |
| **diagnosis*sector** | **1** | **0** | **3** | **0** | -1.91 | 0.28 | 1947 | -6.77 | <.0001 | Tukey-Kramer | <.0001 | 0.05 | -2.46 | -1.36 | -2.99 | -0.82 |
| **diagnosis*sector** | **1** | **0** | **4** | **0** | -2.25 | 0.28 | 1947 | -7.97 | <.0001 | Tukey-Kramer | <.0001 | 0.05 | -2.80 | -1.69 | -3.33 | -1.16 |
| **diagnosis*sector** | **1** | **0** | **9** | **0** | -1.66 | 0.28 | 1947 | -5.89 | <.0001 | Tukey-Kramer | <.0001 | 0.05 | -2.21 | -1.11 | -2.74 | -0.58 |
| **diagnosis*sector** | **10** | **0** | **12** | **0** | 1.51 | 0.28 | 1947 | 5.36 | <.0001 | Tukey-Kramer | <.0001 | 0.05 | 0.96 | 2.07 | 0.43 | 2.60 |
| **diagnosis*sector** | **10** | **0** | **6** | **0** | 1.55 | 0.28 | 1947 | 5.48 | <.0001 | Tukey-Kramer | <.0001 | 0.05 | 0.99 | 2.10 | 0.46 | 2.63 |
| **diagnosis*sector** | **10** | **0** | **7** | **0** | 1.54 | 0.28 | 1947 | 5.46 | <.0001 | Tukey-Kramer | <.0001 | 0.05 | 0.99 | 2.09 | 0.46 | 2.62 |
| **diagnosis*sector** | **11** | **0** | **3** | **0** | -1.85 | 0.28 | 1947 | -6.56 | <.0001 | Tukey-Kramer | <.0001 | 0.05 | -2.40 | -1.30 | -2.93 | -0.77 |
| **diagnosis*sector** | **11** | **0** | **4** | **0** | -2.19 | 0.28 | 1947 | -7.76 | <.0001 | Tukey-Kramer | <.0001 | 0.05 | -2.74 | -1.64 | -3.27 | -1.11 |
| **diagnosis*sector** | **11** | **0** | **9** | **0** | -1.60 | 0.28 | 1947 | -5.69 | <.0001 | Tukey-Kramer | <.0001 | 0.05 | -2.16 | -1.05 | -2.69 | -0.52 |
| **diagnosis*sector** | **12** | **0** | **3** | **0** | -2.00 | 0.28 | 1947 | -7.10 | <.0001 | Tukey-Kramer | <.0001 | 0.05 | -2.56 | -1.45 | -3.09 | -0.92 |
| **diagnosis*sector** | **12** | **0** | **4** | **0** | -2.34 | 0.28 | 1947 | -8.30 | <.0001 | Tukey-Kramer | <.0001 | 0.05 | -2.89 | -1.79 | -3.42 | -1.26 |
| **diagnosis*sector** | **12** | **0** | **9** | **0** | -1.76 | 0.28 | 1947 | -6.23 | <.0001 | Tukey-Kramer | <.0001 | 0.05 | -2.31 | -1.20 | -2.84 | -0.67 |
| **diagnosis*sector** | **2** | **0** | **3** | **0** | -1.79 | 0.28 | 1947 | -6.35 | <.0001 | Tukey-Kramer | <.0001 | 0.05 | -2.34 | -1.24 | -2.87 | -0.71 |
| **diagnosis*sector** | **2** | **0** | **4** | **0** | -2.13 | 0.28 | 1947 | -7.55 | <.0001 | Tukey-Kramer | <.0001 | 0.05 | -2.68 | -1.58 | -3.21 | -1.05 |
| **diagnosis*sector** | **2** | **0** | **9** | **0** | -1.54 | 0.28 | 1947 | -5.47 | <.0001 | Tukey-Kramer | <.0001 | 0.05 | -2.10 | -0.99 | -2.63 | -0.46 |
| **diagnosis*sector** | **3** | **0** | **5** | **0** | 1.56 | 0.28 | 1947 | 5.53 | <.0001 | Tukey-Kramer | <.0001 | 0.05 | 1.01 | 2.11 | 0.48 | 2.64 |
| **diagnosis*sector** | **3** | **0** | **6** | **0** | 2.04 | 0.28 | 1947 | 7.22 | <.0001 | Tukey-Kramer | <.0001 | 0.05 | 1.48 | 2.59 | 0.95 | 3.12 |
| **diagnosis*sector** | **3** | **0** | **7** | **0** | 2.03 | 0.28 | 1947 | 7.20 | <.0001 | Tukey-Kramer | <.0001 | 0.05 | 1.48 | 2.58 | 0.95 | 3.11 |
| **diagnosis*sector** | **3** | **0** | **8** | **0** | 1.78 | 0.28 | 1947 | 6.32 | <.0001 | Tukey-Kramer | <.0001 | 0.05 | 1.23 | 2.34 | 0.70 | 2.87 |
| **diagnosis*sector** | **3** | **0** | **6** | **2** | 4.78 | 0.90 | 1947 | 5.32 | <.0001 | Tukey-Kramer | <.0001 | 0.05 | 3.01 | 6.54 | 1.33 | 8.23 |
| **diagnosis*sector** | **4** | **0** | **5** | **0** | 1.90 | 0.28 | 1947 | 6.73 | <.0001 | Tukey-Kramer | <.0001 | 0.05 | 1.34 | 2.45 | 0.81 | 2.98 |
| **diagnosis*sector** | **4** | **0** | **6** | **0** | 2.37 | 0.28 | 1947 | 8.42 | <.0001 | Tukey-Kramer | <.0001 | 0.05 | 1.82 | 2.93 | 1.29 | 3.46 |
| **diagnosis*sector** | **4** | **0** | **7** | **0** | 2.37 | 0.28 | 1947 | 8.40 | <.0001 | Tukey-Kramer | <.0001 | 0.05 | 1.82 | 2.92 | 1.29 | 3.45 |
| **diagnosis*sector** | **4** | **0** | **8** | **0** | 2.12 | 0.28 | 1947 | 7.52 | <.0001 | Tukey-Kramer | <.0001 | 0.05 | 1.57 | 2.67 | 1.04 | 3.20 |
| **diagnosis*sector** | **4** | **0** | **6** | **2** | 5.11 | 0.90 | 1947 | 5.70 | <.0001 | Tukey-Kramer | <.0001 | 0.05 | 3.35 | 6.87 | 1.66 | 8.56 |
| **diagnosis*sector** | **6** | **0** | **9** | **0** | -1.79 | 0.28 | 1947 | -6.35 | <.0001 | Tukey-Kramer | <.0001 | 0.05 | -2.34 | -1.24 | -2.87 | -0.71 |
| **diagnosis*sector** | **7** | **0** | **9** | **0** | -1.78 | 0.28 | 1947 | -6.33 | <.0001 | Tukey-Kramer | <.0001 | 0.05 | -2.34 | -1.23 | -2.87 | -0.70 |
| **diagnosis*sector** | **8** | **0** | **9** | **0** | -1.54 | 0.28 | 1947 | -5.45 | <.0001 | Tukey-Kramer | <.0001 | 0.05 | -2.09 | -0.98 | -2.62 | -0.45 |
| **diagnosis*sector** | **4** | **1** | **6** | **1** | 2.96 | 0.51 | 1947 | 5.87 | <.0001 | Tukey-Kramer | <.0001 | 0.05 | 1.97 | 3.95 | 1.02 | 4.91 |
| **diagnosis*sector** | **6** | **1** | **9** | **1** | -2.79 | 0.51 | 1947 | -5.52 | <.0001 | Tukey-Kramer | <.0001 | 0.05 | -3.78 | -1.80 | -4.73 | -0.85 |
| **diagnosis*sector** | **1** | **2** | **3** | **2** | -3.80 | 0.70 | 1947 | -5.46 | <.0001 | Tukey-Kramer | <.0001 | 0.05 | -5.17 | -2.44 | -6.48 | -1.12 |
| **diagnosis*sector** | **1** | **2** | **4** | **2** | -4.21 | 0.70 | 1947 | -6.05 | <.0001 | Tukey-Kramer | <.0001 | 0.05 | -5.58 | -2.85 | -6.89 | -1.54 |
| **diagnosis*sector** | **11** | **2** | **4** | **2** | -3.82 | 0.70 | 1947 | -5.48 | <.0001 | Tukey-Kramer | <.0001 | 0.05 | -5.18 | -2.45 | -6.49 | -1.14 |
| **diagnosis*sector** | **12** | **2** | **4** | **2** | -4.04 | 0.70 | 1947 | -5.80 | <.0001 | Tukey-Kramer | <.0001 | 0.05 | -5.41 | -2.67 | -6.72 | -1.36 |
| **diagnosis*sector** | **3** | **2** | **6** | **2** | 5.06 | 0.70 | 1947 | 7.27 | <.0001 | Tukey-Kramer | <.0001 | 0.05 | 3.70 | 6.43 | 2.39 | 7.74 |
| **diagnosis*sector** | **3** | **2** | **7** | **2** | 4.62 | 0.70 | 1947 | 6.64 | <.0001 | Tukey-Kramer | <.0001 | 0.05 | 3.26 | 5.99 | 1.95 | 7.30 |
| **diagnosis*sector** | **3** | **2** | **8** | **2** | 4.38 | 0.70 | 1947 | 6.29 | <.0001 | Tukey-Kramer | <.0001 | 0.05 | 3.02 | 5.75 | 1.71 | 7.06 |
| **diagnosis*sector** | **4** | **2** | **6** | **2** | 5.47 | 0.70 | 1947 | 7.86 | <.0001 | Tukey-Kramer | <.0001 | 0.05 | 4.11 | 6.84 | 2.80 | 8.15 |
| **diagnosis*sector** | **4** | **2** | **7** | **2** | 5.04 | 0.70 | 1947 | 7.23 | <.0001 | Tukey-Kramer | <.0001 | 0.05 | 3.67 | 6.40 | 2.36 | 7.71 |
| **diagnosis*sector** | **4** | **2** | **8** | **2** | 4.79 | 0.70 | 1947 | 6.88 | <.0001 | Tukey-Kramer | <.0001 | 0.05 | 3.43 | 6.16 | 2.12 | 7.47 |
| **diagnosis*sector** | **6** | **2** | **9** | **2** | -3.74 | 0.70 | 1947 | -5.37 | <.0001 | Tukey-Kramer | <.0001 | 0.05 | -5.11 | -2.38 | -6.42 | -1.07 |
| **diagnosis*sector** | **4** | **0** | **7** | **2** | 4.68 | 0.90 | 1947 | 5.21 | <.0001 | Tukey-Kramer | 0.0001 | 0.05 | 2.92 | 6.44 | 1.23 | 8.13 |

C)

| **DCP** | | | | | | | | | | | | | | | | |
| --- | --- | --- | --- | --- | --- | --- | --- | --- | --- | --- | --- | --- | --- | --- | --- | --- |
| **Differences of Least Squares Means** | | | | | | | | | | | | | | | | |
| **Effect** | **Sector** | **Glk** | **Sector** | **Glk** | **Estimate** | **Standard** | **DF** | **t Value** | **Pr > \|t\|** | **Adjustment** | **Adj P** | **Alpha** | **Lower** | **Upper** | **Adj Lower** | **Adj Upper** |
|  |  |  |  |  |  | **Error** |  |  |  |  |  |  |  |  |  |  |
| **sector** | **2** |  | **3** |  | -1.73 | 0.38 | 1947 | -4.56 | <.0001 | Tukey-Kramer | 0.0003 | 0.05 | -2.47 | -0.98 | -2.97 | -0.49 |
| **sector** | **2** |  | **7** |  | 1.74 | 0.38 | 1947 | 4.59 | <.0001 | Tukey-Kramer | 0.0003 | 0.05 | 1.00 | 2.48 | 0.50 | 2.98 |
| **sector** | **2** |  | **8** |  | 1.75 | 0.38 | 1947 | 4.6 | <.0001 | Tukey-Kramer | 0.0003 | 0.05 | 1.00 | 2.49 | 0.50 | 2.99 |
| **sector** | **2** |  | **6** |  | 1.59 | 0.38 | 1947 | 4.19 | <.0001 | Tukey-Kramer | 0.0017 | 0.05 | 0.84 | 2.33 | 0.35 | 2.83 |
| **sector** | **8** |  | **9** |  | -1.44 | 0.38 | 1947 | -3.8 | 0.0001 | Tukey-Kramer | 0.0081 | 0.05 | -2.19 | -0.70 | -2.68 | -0.20 |
| **sector** | **7** |  | **9** |  | -1.44 | 0.38 | 1947 | -3.79 | 0.0002 | Tukey-Kramer | 0.0085 | 0.05 | -2.18 | -0.69 | -2.68 | -0.20 |
| **sector** | **1** |  | **8** |  | 1.40 | 0.38 | 1947 | 3.7 | 0.0002 | Tukey-Kramer | 0.0118 | 0.05 | 0.66 | 2.15 | 0.16 | 2.64 |
| **sector** | **1** |  | **7** |  | 1.40 | 0.38 | 1947 | 3.69 | 0.0002 | Tukey-Kramer | 0.0122 | 0.05 | 0.66 | 2.14 | 0.16 | 2.64 |
| **sector** | **11** |  | **2** |  | -1.30 | 0.38 | 1947 | -3.42 | 0.0006 | Tukey-Kramer | 0.0312 | 0.05 | -2.04 | -0.55 | -2.54 | -0.06 |
| **sector** | **6** |  | **9** |  | -1.29 | 0.38 | 1947 | -3.39 | 0.0007 | Tukey-Kramer | 0.0345 | 0.05 | -2.03 | -0.54 | -2.53 | -0.04 |
| **sector** | **1** |  | **6** |  | 1.25 | 0.38 | 1947 | 3.29 | 0.001 | Tukey-Kramer | 0.0472 | 0.05 | 0.50 | 1.99 | 0.01 | 2.49 |
| **sector** | **1** |  | **3** |  | -2.07 | 0.38 | 1947 | -5.46 | <.0001 | Tukey-Kramer | <.0001 | 0.05 | -2.81 | -1.33 | -3.31 | -0.83 |
| **sector** | **1** |  | **4** |  | -2.32 | 0.38 | 1947 | -6.13 | <.0001 | Tukey-Kramer | <.0001 | 0.05 | -3.07 | -1.58 | -3.57 | -1.08 |
| **sector** | **10** |  | **3** |  | -2.39 | 0.38 | 1947 | -6.3 | <.0001 | Tukey-Kramer | <.0001 | 0.05 | -3.13 | -1.64 | -3.63 | -1.15 |
| **sector** | **10** |  | **4** |  | -2.64 | 0.38 | 1947 | -6.97 | <.0001 | Tukey-Kramer | <.0001 | 0.05 | -3.39 | -1.90 | -3.88 | -1.40 |
| **sector** | **11** |  | **3** |  | -3.03 | 0.38 | 1947 | -7.98 | <.0001 | Tukey-Kramer | <.0001 | 0.05 | -3.77 | -2.28 | -4.27 | -1.78 |
| **sector** | **11** |  | **4** |  | -3.28 | 0.38 | 1947 | -8.65 | <.0001 | Tukey-Kramer | <.0001 | 0.05 | -4.02 | -2.54 | -4.52 | -2.04 |
| **sector** | **12** |  | **3** |  | -2.58 | 0.38 | 1947 | -6.8 | <.0001 | Tukey-Kramer | <.0001 | 0.05 | -3.32 | -1.83 | -3.82 | -1.34 |
| **sector** | **12** |  | **4** |  | -2.83 | 0.38 | 1947 | -7.47 | <.0001 | Tukey-Kramer | <.0001 | 0.05 | -3.58 | -2.09 | -4.07 | -1.59 |
| **sector** | **2** |  | **4** |  | -1.98 | 0.38 | 1947 | -5.23 | <.0001 | Tukey-Kramer | <.0001 | 0.05 | -2.73 | -1.24 | -3.22 | -0.74 |
| **sector** | **3** |  | **5** |  | 2.46 | 0.38 | 1947 | 6.49 | <.0001 | Tukey-Kramer | <.0001 | 0.05 | 1.72 | 3.21 | 1.22 | 3.70 |
| **sector** | **3** |  | **6** |  | 3.32 | 0.38 | 1947 | 8.75 | <.0001 | Tukey-Kramer | <.0001 | 0.05 | 2.57 | 4.06 | 2.08 | 4.56 |
| **sector** | **3** |  | **7** |  | 3.47 | 0.38 | 1947 | 9.15 | <.0001 | Tukey-Kramer | <.0001 | 0.05 | 2.73 | 4.21 | 2.23 | 4.71 |
| **sector** | **3** |  | **8** |  | 3.47 | 0.38 | 1947 | 9.16 | <.0001 | Tukey-Kramer | <.0001 | 0.05 | 2.73 | 4.22 | 2.23 | 4.71 |
| **sector** | **3** |  | **9** |  | 2.03 | 0.38 | 1947 | 5.36 | <.0001 | Tukey-Kramer | <.0001 | 0.05 | 1.29 | 2.77 | 0.79 | 3.27 |
| **sector** | **4** |  | **5** |  | 2.72 | 0.38 | 1947 | 7.17 | <.0001 | Tukey-Kramer | <.0001 | 0.05 | 1.98 | 3.46 | 1.48 | 3.96 |
| **sector** | **4** |  | **6** |  | 3.57 | 0.38 | 1947 | 9.42 | <.0001 | Tukey-Kramer | <.0001 | 0.05 | 2.83 | 4.32 | 2.33 | 4.81 |
| **sector** | **4** |  | **7** |  | 3.72 | 0.38 | 1947 | 9.82 | <.0001 | Tukey-Kramer | <.0001 | 0.05 | 2.98 | 4.47 | 2.48 | 4.97 |
| **sector** | **4** |  | **8** |  | 3.73 | 0.38 | 1947 | 9.83 | <.0001 | Tukey-Kramer | <.0001 | 0.05 | 2.99 | 4.47 | 2.49 | 4.97 |
| **sector** | **4** |  | **9** |  | 2.29 | 0.38 | 1947 | 6.03 | <.0001 | Tukey-Kramer | <.0001 | 0.05 | 1.54 | 3.03 | 1.05 | 3.53 |
| **diagnosis*sector** | **3** | **1** | **6** | **1** | 3.24 | 0.63 | 1947 | 5.11 | <.0001 | Tukey-Kramer | 0.0002 | 0.05 | 2.00 | 4.49 | 0.80 | 5.68 |
| **diagnosis*sector** | **4** | **1** | **7** | **1** | 3.18 | 0.63 | 1947 | 5.01 | <.0001 | Tukey-Kramer | 0.0003 | 0.05 | 1.94 | 4.43 | 0.74 | 5.62 |
| **diagnosis*sector** | **4** | **0** | **6** | **0** | 1.77 | 0.35 | 1947 | 4.99 | <.0001 | Tukey-Kramer | 0.0004 | 0.05 | 1.07 | 2.46 | 0.41 | 3.13 |
| **diagnosis*sector** | **10** | **2** | **4** | **2** | -4.36 | 0.88 | 1947 | -4.98 | <.0001 | Tukey-Kramer | 0.0004 | 0.05 | -6.08 | -2.64 | -7.72 | -1.00 |
| **diagnosis*sector** | **3** | **0** | **6** | **0** | 1.72 | 0.35 | 1947 | 4.86 | <.0001 | Tukey-Kramer | 0.0007 | 0.05 | 1.03 | 2.42 | 0.36 | 3.08 |
| **diagnosis*sector** | **12** | **2** | **3** | **2** | -4.24 | 0.88 | 1947 | -4.85 | <.0001 | Tukey-Kramer | 0.0008 | 0.05 | -5.96 | -2.53 | -7.60 | -0.88 |
| **diagnosis*sector** | **6** | **1** | **4** | **2** | -5.39 | 1.13 | 1947 | -4.77 | <.0001 | Tukey-Kramer | 0.0011 | 0.05 | -7.60 | -3.17 | -9.73 | -1.05 |
| **diagnosis*sector** | **10** | **2** | **3** | **2** | -4.06 | 0.88 | 1947 | -4.63 | <.0001 | Tukey-Kramer | 0.0021 | 0.05 | -5.77 | -2.34 | -7.42 | -0.69 |
| **diagnosis*sector** | **4** | **0** | **8** | **2** | 4.98 | 1.10 | 1947 | 4.54 | <.0001 | Tukey-Kramer | 0.0032 | 0.05 | 2.83 | 7.12 | 0.76 | 9.19 |
| **diagnosis*sector** | **11** | **1** | **3** | **1** | -2.87 | 0.63 | 1947 | -4.52 | <.0001 | Tukey-Kramer | 0.0035 | 0.05 | -4.11 | -1.62 | -5.31 | -0.43 |
| **diagnosis*sector** | **6** | **1** | **3** | **2** | -5.08 | 1.13 | 1947 | -4.5 | <.0001 | Tukey-Kramer | 0.0037 | 0.05 | -7.30 | -2.87 | -9.42 | -0.75 |
| **diagnosis*sector** | **3** | **0** | **8** | **2** | 4.93 | 1.10 | 1947 | 4.5 | <.0001 | Tukey-Kramer | 0.0038 | 0.05 | 2.78 | 7.08 | 0.72 | 9.14 |
| **diagnosis*sector** | **4** | **2** | **5** | **2** | 3.93 | 0.88 | 1947 | 4.5 | <.0001 | Tukey-Kramer | 0.0038 | 0.05 | 2.22 | 5.65 | 0.57 | 7.30 |
| **diagnosis*sector** | **4** | **0** | **6** | **1** | 3.99 | 0.90 | 1947 | 4.44 | <.0001 | Tukey-Kramer | 0.0049 | 0.05 | 2.23 | 5.75 | 0.53 | 7.44 |
| **diagnosis*sector** | **11** | **1** | **4** | **2** | -5.01 | 1.13 | 1947 | -4.44 | <.0001 | Tukey-Kramer | 0.0049 | 0.05 | -7.23 | -2.80 | -9.35 | -0.67 |
| **diagnosis*sector** | **11** | **0** | **4** | **0** | -1.56 | 0.35 | 1947 | -4.4 | <.0001 | Tukey-Kramer | 0.0057 | 0.05 | -2.25 | -0.87 | -2.92 | -0.20 |
| **diagnosis*sector** | **3** | **0** | **6** | **1** | 3.94 | 0.90 | 1947 | 4.39 | <.0001 | Tukey-Kramer | 0.0061 | 0.05 | 2.18 | 5.71 | 0.49 | 7.40 |
| **diagnosis*sector** | **3** | **1** | **7** | **1** | 2.76 | 0.63 | 1947 | 4.35 | <.0001 | Tukey-Kramer | 0.0071 | 0.05 | 1.52 | 4.01 | 0.32 | 5.20 |
| **diagnosis*sector** | **4** | **1** | **5** | **1** | 2.76 | 0.63 | 1947 | 4.35 | <.0001 | Tukey-Kramer | 0.0072 | 0.05 | 1.52 | 4.01 | 0.32 | 5.20 |
| **diagnosis*sector** | **7** | **1** | **4** | **2** | -4.91 | 1.13 | 1947 | -4.35 | <.0001 | Tukey-Kramer | 0.0073 | 0.05 | -7.12 | -2.69 | -9.25 | -0.57 |
| **diagnosis*sector** | **4** | **2** | **9** | **2** | 3.75 | 0.88 | 1947 | 4.29 | <.0001 | Tukey-Kramer | 0.0094 | 0.05 | 2.03 | 5.47 | 0.39 | 7.11 |
| **diagnosis*sector** | **11** | **0** | **3** | **0** | -1.52 | 0.35 | 1947 | -4.28 | <.0001 | Tukey-Kramer | 0.0097 | 0.05 | -2.21 | -0.82 | -2.88 | -0.15 |
| **diagnosis*sector** | **1** | **2** | **4** | **2** | -3.74 | 0.88 | 1947 | -4.27 | <.0001 | Tukey-Kramer | 0.01 | 0.05 | -5.45 | -2.02 | -7.10 | -0.37 |
| **diagnosis*sector** | **12** | **1** | **4** | **1** | -2.65 | 0.63 | 1947 | -4.18 | <.0001 | Tukey-Kramer | 0.0145 | 0.05 | -3.90 | -1.41 | -5.09 | -0.21 |
| **diagnosis*sector** | **11** | **1** | **3** | **2** | -4.71 | 1.13 | 1947 | -4.17 | <.0001 | Tukey-Kramer | 0.015 | 0.05 | -6.92 | -2.49 | -9.05 | -0.37 |
| **diagnosis*sector** | **3** | **2** | **5** | **2** | 3.63 | 0.88 | 1947 | 4.15 | <.0001 | Tukey-Kramer | 0.0162 | 0.05 | 1.91 | 5.35 | 0.27 | 6.99 |
| **diagnosis*sector** | **4** | **0** | **5** | **0** | 1.46 | 0.35 | 1947 | 4.12 | <.0001 | Tukey-Kramer | 0.0182 | 0.05 | 0.76 | 2.15 | 0.10 | 2.82 |
| **diagnosis*sector** | **4** | **0** | **7** | **2** | 4.51 | 1.10 | 1947 | 4.12 | <.0001 | Tukey-Kramer | 0.0183 | 0.05 | 2.36 | 6.66 | 0.30 | 8.72 |
| **diagnosis*sector** | **4** | **1** | **8** | **2** | 4.65 | 1.13 | 1947 | 4.12 | <.0001 | Tukey-Kramer | 0.0183 | 0.05 | 2.44 | 6.86 | 0.31 | 8.99 |
| **diagnosis*sector** | **3** | **0** | **7** | **2** | 4.47 | 1.10 | 1947 | 4.08 | <.0001 | Tukey-Kramer | 0.0214 | 0.05 | 2.32 | 6.62 | 0.26 | 8.68 |
| **diagnosis*sector** | **7** | **1** | **3** | **2** | -4.60 | 1.13 | 1947 | -4.08 | <.0001 | Tukey-Kramer | 0.0214 | 0.05 | -6.82 | -2.39 | -8.94 | -0.26 |
| **diagnosis*sector** | **4** | **1** | **8** | **1** | 2.59 | 0.63 | 1947 | 4.07 | <.0001 | Tukey-Kramer | 0.0216 | 0.05 | 1.34 | 3.83 | 0.15 | 5.03 |
| **diagnosis*sector** | **4** | **0** | **11** | **1** | 3.61 | 0.90 | 1947 | 4.02 | <.0001 | Tukey-Kramer | 0.0267 | 0.05 | 1.85 | 5.38 | 0.16 | 7.07 |
| **diagnosis*sector** | **3** | **0** | **5** | **0** | 1.41 | 0.35 | 1947 | 3.99 | <.0001 | Tukey-Kramer | 0.0293 | 0.05 | 0.72 | 2.11 | 0.05 | 2.78 |
| **diagnosis*sector** | **5** | **1** | **4** | **2** | -4.49 | 1.13 | 1947 | -3.97 | <.0001 | Tukey-Kramer | 0.0314 | 0.05 | -6.70 | -2.27 | -8.83 | -0.15 |
| **diagnosis*sector** | **3** | **0** | **11** | **1** | 3.57 | 0.90 | 1947 | 3.97 | <.0001 | Tukey-Kramer | 0.0321 | 0.05 | 1.80 | 5.33 | 0.11 | 7.02 |
| **diagnosis*sector** | **3** | **2** | **9** | **2** | 3.45 | 0.88 | 1947 | 3.94 | <.0001 | Tukey-Kramer | 0.0358 | 0.05 | 1.73 | 5.16 | 0.08 | 6.81 |
| **diagnosis*sector** | **2** | **2** | **8** | **2** | 3.44 | 0.88 | 1947 | 3.93 | <.0001 | Tukey-Kramer | 0.0363 | 0.05 | 1.73 | 5.16 | 0.08 | 6.81 |
| **diagnosis*sector** | **10** | **0** | **4** | **0** | -1.39 | 0.35 | 1947 | -3.93 | <.0001 | Tukey-Kramer | 0.0373 | 0.05 | -2.09 | -0.70 | -2.75 | -0.03 |
| **diagnosis*sector** | **1** | **2** | **3** | **2** | -3.43 | 0.88 | 1947 | -3.92 | <.0001 | Tukey-Kramer | 0.0377 | 0.05 | -5.15 | -1.72 | -6.80 | -0.07 |
| **diagnosis*sector** | **4** | **0** | **7** | **1** | 3.51 | 0.90 | 1947 | 3.9 | <.0001 | Tukey-Kramer | 0.0406 | 0.05 | 1.75 | 5.27 | 0.05 | 6.96 |
| **diagnosis*sector** | **12** | **2** | **4** | **2** | -4.38 | 1.13 | 1947 | -3.88 | 0.0001 | Tukey-Kramer | 0.0445 | 0.05 | -6.59 | -2.16 | -8.72 | -0.04 |
| **diagnosis*sector** | **3** | **0** | **7** | **1** | 3.46 | 0.90 | 1947 | 3.85 | 0.0001 | Tukey-Kramer | 0.0483 | 0.05 | 1.70 | 5.23 | 0.01 | 6.92 |
| **diagnosis*sector** | **3** | **0** | **7** | **0** | 2.04 | 0.35 | 1947 | 5.75 | <.0001 | Tukey-Kramer | <.0001 | 0.05 | 1.34 | 2.73 | 0.67 | 3.40 |
| **diagnosis*sector** | **3** | **0** | **8** | **0** | 2.18 | 0.35 | 1947 | 6.16 | <.0001 | Tukey-Kramer | <.0001 | 0.05 | 1.49 | 2.88 | 0.82 | 3.54 |
| **diagnosis*sector** | **4** | **0** | **7** | **0** | 2.08 | 0.35 | 1947 | 5.87 | <.0001 | Tukey-Kramer | <.0001 | 0.05 | 1.39 | 2.78 | 0.72 | 3.44 |
| **diagnosis*sector** | **4** | **0** | **8** | **0** | 2.23 | 0.35 | 1947 | 6.28 | <.0001 | Tukey-Kramer | <.0001 | 0.05 | 1.53 | 2.92 | 0.86 | 3.59 |
| **diagnosis*sector** | **4** | **1** | **6** | **1** | 3.66 | 0.63 | 1947 | 5.77 | <.0001 | Tukey-Kramer | <.0001 | 0.05 | 2.42 | 4.91 | 1.22 | 6.10 |
| **diagnosis*sector** | **11** | **2** | **3** | **2** | -4.69 | 0.88 | 1947 | -5.36 | <.0001 | Tukey-Kramer | <.0001 | 0.05 | -6.41 | -2.98 | -8.06 | -1.33 |
| **diagnosis*sector** | **11** | **2** | **4** | **2** | -5.00 | 0.88 | 1947 | -5.71 | <.0001 | Tukey-Kramer | <.0001 | 0.05 | -6.71 | -3.28 | -8.36 | -1.63 |
| **diagnosis*sector** | **3** | **2** | **6** | **2** | 4.98 | 0.88 | 1947 | 5.69 | <.0001 | Tukey-Kramer | <.0001 | 0.05 | 3.27 | 6.70 | 1.62 | 8.34 |
| **diagnosis*sector** | **3** | **2** | **7** | **2** | 5.61 | 0.88 | 1947 | 6.41 | <.0001 | Tukey-Kramer | <.0001 | 0.05 | 3.89 | 7.32 | 2.25 | 8.97 |
| **diagnosis*sector** | **3** | **2** | **8** | **2** | 6.07 | 0.88 | 1947 | 6.94 | <.0001 | Tukey-Kramer | <.0001 | 0.05 | 4.35 | 7.79 | 2.71 | 9.43 |
| **diagnosis*sector** | **4** | **2** | **6** | **2** | 5.29 | 0.88 | 1947 | 6.04 | <.0001 | Tukey-Kramer | <.0001 | 0.05 | 3.57 | 7.00 | 1.92 | 8.65 |
| **diagnosis*sector** | **4** | **2** | **7** | **2** | 5.91 | 0.88 | 1947 | 6.76 | <.0001 | Tukey-Kramer | <.0001 | 0.05 | 4.20 | 7.63 | 2.55 | 9.27 |
| **diagnosis*sector** | **4** | **2** | **8** | **2** | 6.37 | 0.88 | 1947 | 7.29 | <.0001 | Tukey-Kramer | <.0001 | 0.05 | 4.66 | 8.09 | 3.01 | 9.74 |
| **diagnosis*sector** | **11** | **1** | **4** | **1** | -3.29 | 0.63 | 1947 | -5.18 | <.0001 | Tukey-Kramer | 0.0001 | 0.05 | -4.53 | -2.04 | -5.73 | -0.85 |
| **diagnosis*sector** | **12** | **2** | **4** | **2** | -4.55 | 0.88 | 1947 | -5.19 | <.0001 | Tukey-Kramer | 0.0001 | 0.05 | -6.26 | -2.83 | -7.91 | -1.18 |
